# Supplementary material for: Sexually transmitted and blood-borne infections in transgender and non-binary people in Canada: A scoping review
Source: PLoS One. 2025 May 20;20(5):e0322521. doi: 10.1371/journal.pone.0322521 (PMC12091819; doi:10.1371/journal.pone.0322521)
Supplement: S1 File — (DOCX) [file pone.0322521.s001.docx]

**S1 Supporting Information: Details of database and grey literature searches**

NUMBER OF RESULTS

| Medline | Embase | CAB Abstracts and Global Health | PsycInfo | Scopus | All results prior to removing duplicates | All results after removing duplicates | Duplicates removed |
| --- | --- | --- | --- | --- | --- | --- | --- |
| 122 | 226 | 101 | 57 | 169 | 675 | **381** | 294 |

SEARCH STRATEGY

## MEDLINE

Database(s): **Ovid MEDLINE(R) ALL**1946 to September 01, 2023
Search Strategy:

| **#** | **Searches** | **Results** |
| --- | --- | --- |
| 1 | exp sexually transmitted diseases/ | 409604 |
| 2 | ((("sexually transmitted" or Venereal) adj2 (disease* or infect*)) or STBBI? or blood borne infection? or STDs or STD or STI or STIs).tw,kf. | 56086 |
| 3 | exp HIV/ or hiv antibodies/ or (hiv or hiv-1 or hiv-2 or hiv1 or hiv2 or hivaids or human immune deficiency virus or human immunodeficiency virus or human immuno-deficiency virus or human immune-deficiency virus or acquired immunodeficiency or acquired immune deficiency or acquired immuno-deficiency).tw,kf,kw. | 395738 |
| 4 | exp herpes simplex/ or (herpes or "hhv-1" or "hsv-1" or "hhv-2" or "hsv-2" or "herpesvirus" or herpes simplex virus or HSV or Kaposi Varicelliform Eruption or Herpetic Keratitis or Dendritic Keratitis or Herpetic Stomatitis or Herpetic Stomatitides or Eczema Vaccinatum or Eczema Herpeticum or Herpetic Keratitides or Dendritic Keratitides or Furrow Keratitis or Furrow Keratitides).tw,kf,kw. | 99247 |
| 5 | exp Human Papillomavirus Viruses/ or (((condyloma or condyla or condylatum or condylomata) adj3 (acuminatum or acuminata)) or verruca accuminata or vulvar condyloma or hpv or "human papillomavirus" or "Human Papilloma Virus" or ((genital or venereal or penile or perianal or anal or anogenital) adj3 wart*) or bowenoid papulosis or bowen dermatosis or bowenoid lesion or bowen disease?).tw,kf. | 67027 |
| 6 | hepatitis a/ or exp Hepatitis A virus/ or Hepatitis A Antibodies/ or Hepatitis A Antigens/ or Hepatitis A Vaccines/ or exp hepatitis b/ or exp Hepatitis B virus/ or Hepatitis B Antibodies/ or Hepatitis B Antigens/ or Hepatitis B Vaccines/ or exp hepatitis c/ or exp Hepatitis C virus/ or Hepatitis C Antibodies/ or Hepatitis C Antigens/ or Hepatitis C Vaccines/ or ("hep c" or hepatitis c or "hcv" or "hep b" or hepatitis b or "hbv" or "hep a" or hepatitis a or "hav").tw,kf,kw. | 219595 |
| 7 | neisseria gonorrhoeae/ or (gonorrhea or gonococcus or gonorrhoeae or gonorrhoea or gonococci or gonococcal or gonococcosis).tw,kf,kw. | 26103 |
| 8 | chlamydia/ or Chlamydia trachomatis/ or (chlamydi* or "c trachomatis" or C trachomatis or Chlamydiaceae* or Chlamydophila* or Lymphogranuloma Venereum or lymphopathia venerea or lymphopathia venereum or lymphogranuloma inguinale or inguinal lymphogranulomatosis or venereal lymphogranuloma or Durand-Nicholas-Favre disease or Favre-Durand disease or Frei disease or Nicholas Favre disease or Nicolas Favre disease or Nicolas-Durand-Favre disease).tw,kw,kf. | 34624 |
| 9 | exp trichomonas infections/ or (trichomoniasis or "trich" or Trichomoniases or Trichomonas).tw,kf,kw. | 11155 |
| 10 | (Granuloma Inguinale or Granuloma Venereum or Donovanosis or granulomatis infection).tw,kf,kw. | 663 |
| 11 | Vaginosis, Bacterial/ or (bacterial vaginosis or bacterial Vaginitides or Bacterial Vaginoses).tw,kf,kw. | 5511 |
| 12 | exp Pelvic Inflammatory Disease/ or (Adnexitis or Pelvic Inflammatory Disease* or Endometritis or Endomyometritis or Oophoritides or Oophoritis or Parametritis or Parametritides or Pelvic Cellulitides or Pelvic Cellulitis or Salpingitis or Salpingitides or pelvis abscess or pelvioperitonitis or metritis or Fitz-Hugh-Curtis syndrome).tw,kf,kw. | 17950 |
| 13 | exp Proctitis/ or (Proctitis or Proctocolitis or Rectocolitis or Ulcerative Rectocolitides or Ulcerative Rectocolitis or Ulcerative Proctocolitis or Proctosigmoiditis or Rectosigmoiditis or rectitis).tw,kf,kw. | 5713 |
| 14 | Phthirus/ or (Phthirus or Crab Louse? or Crab Lice? or pubic lice? or pubic louse? or Pediculus pubis or phthiriasis).tw,kf,kw. | 324 |
| 15 | Scabies/ or (Scabies or Sarcoptic Mange or Sarcoptes scabiei).tw,kf,kw. | 5580 |
| 16 | vaginitis/ or exp Vulvovaginitis/ or (vaginitis or colpitis or kolpitis or Vulvovaginitis or Vaginitides or Vulvovaginitides or Vulvovaginal Candidiasis or Vulvovaginal Moniliasis or Monilial Vaginitis or Genital Candidiasis or (vaginal adj3 yeast)).tw,kf. | 10884 |
| 17 | Urethritis/ or (urethritis or Urethritides).tw,kf,kw. | 6658 |
| 18 | exp HTLV-I Infections/ or HTLV-II Infections/ or Human T-lymphotropic virus 1/ or Human T-lymphotropic virus 2/ or Human T-lymphotropic virus 3/ or (HUMAN T-LYMPHOTROPIC VIRUS or HTLV or ATLV or Adult T-Cell Leukemia-Lymphoma Virus I or Human T-Cell Leukemia Virus I or Adult T Cell Leukemia Lymphoma Virus I or Human T Cell Leukemia Virus I or Human T lymphotropic virus 2 or Human T Lymphotropic Virus II or Human T-Lymphotropic Virus II or Human T Cell Leukemia Virus II or Human T lymphotropic virus 3 or STLV-3).tw,kf,kw. | 15762 |
| 19 | (syphilis* or "great pox" or Chancre or Tabes Dorsalis or neurosyphilis or treponema pallidum or t pallidum or Chancroid* or lues or luetic disease or ulcus molle or Ducrey disease or "Ducrey's streptobacillosis" or ((ducrey or ducreyi) adj2 (Haemophilus or Hemophilus))).tw,kf. | 37547 |
| 20 | Epididymitis/ or epididymitis.tw,kf,kw. | 3642 |
| 21 | Molluscum Contagiosum/ or (molluscum contagiosum or mollusca contagiosa or molluscum epitheliale).tw,kf,kw. | 2238 |
| 22 | or/1-21 [STBBIs] | 954041 |
| 23 | Transgender Persons/ or Transsexualism/ or Sex Reassignment Procedures/ | 10335 |
| 24 | (transsexual* or transgender* or "trans gender*" or transpeople* or transm#n or transwom#n or nonbinary or "non binary" or "two spirit*" or "third gender*" or Transvestite or androgynous or "multi gender*" or "multigender*" or "gender nonconforming" or genderqueer or agender or "female to male" or FTM or "male to female" or MTF).tw,kf,kw. | 36201 |
| 25 | ((trans adj3 (people or individual or individuals or person or persons or sexual* or man or men or male or female or youth* or woman or women or population* or gender* or sex worker* or adult* or patient* or participant* or masc* or femme* or feminine*)) or ((gender* or sex*) adj3 (fluid* or change or dysphoria or reversal or reassign* or transform* or transition*))).tw,kf. | 14692 |
| 26 | ((gender adj3 (neutral* or affirm* or inclusive or responsive or transformative)) or genderless or gender less).tw,kf. | 4205 |
| 27 | or/23-26 [Trans People] | 49565 |
| 28 | exp Canada/ or exp Indigenous Canadians/ or (canada* or canadia* or canadien* or ottawa* or british columbia* or colombie britannique* or vancouver* or alberta* or edmonton* or calgar* or saskatchewan* or regina* or saskatoon* or manitoba* or winnipeg* or ontari* or toronto* or quebec* or montreal* or new brunswick* or nouveau brunswick* or fredericton* or nova scotia* or nouvelle ecosse* or halifax* or haligonian* or prince edward island* or ile du prince edouard* or pei or charlottetown* or newfoundland* or terre neuve* or labrador* or nfld or yukon* or whitehorse* or northwest territor* or territoires du nord ouest* or nwt or yellowknife* or nunavut* or iqaluit*).tw,kf,kw. | 318254 |
| 29 | 22 and 27 and 28 | 154 |
| 30 | limit 29 to yr="2013-Current" | 121 |

## EMBASE

Database(s): **Embase**1974 to 2023 September 01
Search Strategy:

| **#** | **Searches** | **Results** |
| --- | --- | --- |
| 1 | exp sexually transmitted disease/ | 106564 |
| 2 | ((("sexually transmitted" or Venereal) adj2 (disease* or infect*)) or STBBI? or blood borne infection? or STDs or STD or STI or STIs).tw,kf. | 75720 |
| 3 | exp Human immunodeficiency virus infection/ or exp Human immunodeficiency virus/ or Human immunodeficiency virus antigen/ or Human immunodeficiency virus antibody/ or Human immunodeficiency virus infected patient/ or (hiv or hiv-1 or hiv-2 or hiv1 or hiv2 or hivaids or human immune deficiency virus or human immunodeficiency virus or human immuno-deficiency virus or human immune-deficiency virus or acquired immunodeficiency or acquired immune deficiency or acquired immuno-deficiency).tw,kf,kw. | 631514 |
| 4 | exp herpes simplex/ or (herpes or "hhv-1" or "hsv-1" or "hhv-2" or "hsv-2" or "herpesvirus" or herpes simplex virus or HSV or Kaposi Varicelliform Eruption or Herpetic Keratitis or Dendritic Keratitis or Herpetic Stomatitis or Herpetic Stomatitides or Eczema Vaccinatum or Eczema Herpeticum or Herpetic Keratitides or Dendritic Keratitides or Furrow Keratitis or Furrow Keratitides).tw,kf,kw. | 127248 |
| 5 | wart virus/ or papillomavirus infection/ or (((condyloma or condyla or condylatum or condylomata) adj3 (acuminatum or acuminata)) or verruca accuminata or vulvar condyloma or hpv or "human papillomavirus" or "Human Papilloma Virus" or ((genital or venereal or penile or perianal or anal or anogenital) adj3 wart*) or bowenoid papulosis or bowen dermatosis or bowenoid lesion or bowen disease?).tw,kf. | 99579 |
| 6 | exp hepatitis A/ or exp Hepatitis A virus/ or hepatitis A vaccine/ or hepatitis A antibody/ or hepatitis A antigen/ or exp hepatitis B/ or exp Hepatitis B virus/ or hepatitis B vaccine/ or hepatitis B antibody/ or hepatitis B antigen/ or exp hepatitis C/ or exp Hepatitis C virus/ or hepatitis C vaccine/ or hepatitis C antibody/ or hepatitis C antigen/ or ("hep c" or hepatitis c or "hcv" or "hep b" or hepatitis b or "hbv" or "hep a" or hepatitis a or "hav").tw,kf,kw. | 348428 |
| 7 | exp gonorrhea/ or Neisseria gonorrhoeae/ or (gonorrhea or gonococcus or gonorrhoeae or gonorrhoea or gonococci or gonococcal or gonococcosis).tw,kf,kw. | 36662 |
| 8 | exp chlamydia/ or (chlamydi* or "c trachomatis" or C trachomatis or Chlamydiaceae* or Chlamydophila* or Lymphogranuloma Venereum or lymphopathia venerea or lymphopathia venereum or lymphogranuloma inguinale or inguinal lymphogranulomatosis or venereal lymphogranuloma or Durand-Nicholas-Favre disease or Favre-Durand disease or Frei disease or Nicholas Favre disease or Nicolas Favre disease or Nicolas-Durand-Favre disease).tw,kw,kf. | 47288 |
| 9 | exp trichomoniasis/ or (trichomoniasis or "trich" or Trichomoniases or Trichomonas).tw,kf,kw. | 11194 |
| 10 | (Granuloma Inguinale or Granuloma Venereum or Donovanosis or granulomatis infection).tw,kf,kw. | 443 |
| 11 | (bacterial vaginosis or bacterial Vaginitides or Bacterial Vaginoses).tw,kf,kw. | 6552 |
| 12 | exp pelvic inflammatory disease/ or (Adnexitis or Pelvic Inflammatory Disease* or Endometritis or Endomyometritis or Oophoritides or Oophoritis or Parametritis or Parametritides or Pelvic Cellulitides or Pelvic Cellulitis or Salpingitis or Salpingitides or pelvis abscess or pelvioperitonitis or metritis or Fitz-Hugh-Curtis syndrome).tw,kf,kw. | 26607 |
| 13 | exp Proctitis/ or (Proctitis or Proctocolitis or Rectocolitis or Ulcerative Rectocolitides or Ulcerative Rectocolitis or Ulcerative Proctocolitis or Proctosigmoiditis or Rectosigmoiditis or rectitis).tw,kf,kw. | 10552 |
| 14 | Phthirus/ or (Phthirus or Crab Louse? or Crab Lice? or pubic lice? or pubic louse? or Pediculus pubis or phthiriasis).tw,kf,kw. | 376 |
| 15 | Scabies/ or (Scabies or Sarcoptic Mange or Sarcoptes scabiei).tw,kf,kw. | 8054 |
| 16 | exp vaginitis/ or (vaginitis or colpitis or kolpitis or Vulvovaginitis or Vaginitides or Vulvovaginitides or Vulvovaginal Candidiasis or Vulvovaginal Moniliasis or Monilial Vaginitis or Genital Candidiasis or (vaginal adj3 yeast)).tw,kf. | 21305 |
| 17 | exp Urethritis/ or (urethritis or Urethritides).tw,kf,kw. | 8492 |
| 18 | exp human T cell leukemia virus/ or exp Human T cell leukemia virus infection/ or (HUMAN T-LYMPHOTROPIC VIRUS or HTLV or ATLV or Adult T-Cell Leukemia-Lymphoma Virus I or Human T-Cell Leukemia Virus I or Adult T Cell Leukemia Lymphoma Virus I or Human T Cell Leukemia Virus I or Human T lymphotropic virus 2 or Human T Lymphotropic Virus II or Human T-Lymphotropic Virus II or Human T Cell Leukemia Virus II or Human T lymphotropic virus 3 or STLV-3).tw,kf,kw. | 20129 |
| 19 | (syphilis* or "great pox" or Chancre or Tabes Dorsalis or neurosyphilis or treponema pallidum or t pallidum or Chancroid* or lues or luetic disease or ulcus molle or Ducrey disease or "Ducrey's streptobacillosis" or ((ducrey or ducreyi) adj2 (Haemophilus or Hemophilus))).tw,kf. | 32726 |
| 20 | Epididymitis/ or epididymitis.tw,kf,kw. | 5384 |
| 21 | Molluscum Contagiosum/ or (molluscum contagiosum or mollusca contagiosa or molluscum epitheliale).tw,kf,kw. | 3704 |
| 22 | or/1-21 [STBBIs] | 1305355 |
| 23 | exp transgender/ or "gender nonbinary"/ or exp gender dysphoria/ or sex reassignment/ | 19014 |
| 24 | (transsexual* or transgender* or "trans gender*" or transpeople* or transm#n or transwom#n or nonbinary or "non binary" or "two spirit*" or "third gender*" or Transvestite or androgynous or "multi gender*" or "multigender*" or "gender nonconforming" or genderqueer or agender or "female to male" or FTM or "male to female" or MTF).tw,kf,kw. | 52796 |
| 25 | ((trans adj3 (people or individual or individuals or person or persons or sexual* or man or men or male or female or youth* or woman or women or population* or gender* or sex worker* or adult* or patient* or participant* or masc* or femme* or feminine*)) or ((gender* or sex*) adj3 (fluid* or change or dysphoria or reversal or reassign* or transform* or transition*))).tw,kf. | 19741 |
| 26 | ((gender adj3 (neutral* or affirm* or inclusive or responsive or transformative)) or genderless or gender less).tw,kf. | 5226 |
| 27 | or/23-26 [Trans People] | 71654 |
| 28 | exp Canada/ or Canadian/ or Canadian Aboriginal/ or (canada* or canadia* or canadien* or ottawa* or british columbia* or colombie britannique* or vancouver* or alberta* or edmonton* or calgar* or saskatchewan* or regina* or saskatoon* or manitoba* or winnipeg* or ontari* or toronto* or quebec* or montreal* or new brunswick* or nouveau brunswick* or fredericton* or nova scotia* or nouvelle ecosse* or halifax* or haligonian* or prince edward island* or ile du prince edouard* or pei or charlottetown* or newfoundland* or terre neuve* or labrador* or nfld or yukon* or whitehorse* or northwest territor* or territoires du nord ouest* or nwt or yellowknife* or nunavut* or iqaluit*).tw,kf,kw. | 408752 |
| 29 | 22 and 27 and 28 | 290 |
| 30 | limit 29 to yr="2013-Current" | 226 |

## CAB ABSTRACTS AND GLOBAL HEALTH

Accession Number: 20230327071

Database(s): **CAB Abstracts**1973 to 2023 Week 35**, Global Health**1973 to 2023 Week 35
Search Strategy:

| **#** | **Searches** | **Results** |
| --- | --- | --- |
| 1 | exp sexually transmitted diseases/ | 60850 |
| 2 | ((("sexually transmitted" or Venereal) adj2 (disease* or infect*)) or STBBI? or blood borne infection? or STDs or STD or STI or STIs).tw,hw. | 65689 |
| 3 | exp human immunodeficiency viruses/ or (hiv or hiv-1 or hiv-2 or hiv1 or hiv2 or hivaids or human immune deficiency virus or human immunodeficiency virus or human immuno-deficiency virus or human immune-deficiency virus or acquired immunodeficiency or acquired immune deficiency or acquired immuno-deficiency).tw,hw. | 302691 |
| 4 | exp herpes simplex/ or (herpes or "hhv-1" or "hsv-1" or "hhv-2" or "hsv-2" or "herpesvirus" or herpes simplex virus or HSV or Kaposi Varicelliform Eruption or Herpetic Keratitis or Dendritic Keratitis or Herpetic Stomatitis or Herpetic Stomatitides or Eczema Vaccinatum or Eczema Herpeticum or Herpetic Keratitides or Dendritic Keratitides or Furrow Keratitis or Furrow Keratitides).tw,hw. | 79173 |
| 5 | human papillomaviruses/ or (((condyloma or condyla or condylatum or condylomata) adj3 (acuminatum or acuminata)) or verruca accuminata or vulvar condyloma or hpv or "human papillomavirus" or "Human Papilloma Virus" or ((genital or venereal or penile or perianal or anal or anogenital) adj3 wart*) or bowenoid papulosis or bowen dermatosis or bowenoid lesion or bowen disease?).tw,hw. | 32397 |
| 6 | exp Hepatovirus A/ or exp Hepatitis B virus/ or exp Hepatitis C virus/ or ("hep c" or hepatitis c or "hcv" or "hep b" or hepatitis b or "hbv" or "hep a" or hepatitis a or "hav").tw,hw. | 100677 |
| 7 | exp Neisseria gonorrhoeae/ or (gonorrhea or gonococcus or gonorrhoeae or gonorrhoea or gonococci or gonococcal or gonococcosis).tw,hw. | 13847 |
| 8 | chlamydia/ or exp Chlamydia trachomatis/ or (chlamydi* or "c trachomatis" or C trachomatis or Chlamydiaceae* or Chlamydophila* or Lymphogranuloma Venereum or lymphopathia venerea or lymphopathia venereum or lymphogranuloma inguinale or inguinal lymphogranulomatosis or venereal lymphogranuloma or Durand-Nicholas-Favre disease or Favre-Durand disease or Frei disease or Nicholas Favre disease or Nicolas Favre disease or Nicolas-Durand-Favre disease).tw,hw. | 27489 |
| 9 | exp Trichomonas/ or (trichomoniasis or "trich" or Trichomoniases or Trichomonas).tw,hw. | 15486 |
| 10 | (Granuloma Inguinale or Granuloma Venereum or Donovanosis or granulomatis infection).tw,hw. | 236 |
| 11 | (bacterial vaginosis or bacterial Vaginitides or Bacterial Vaginoses).tw,hw. | 4515 |
| 12 | exp Pelvic Inflammatory Disease/ or (Adnexitis or Pelvic Inflammatory Disease* or Endometritis or Endomyometritis or Oophoritides or Oophoritis or Parametritis or Parametritides or Pelvic Cellulitides or Pelvic Cellulitis or Salpingitis or Salpingitides or pelvis abscess or pelvioperitonitis or metritis or Fitz-Hugh-Curtis syndrome).tw,hw. | 12132 |
| 13 | exp Proctitis/ or (Proctitis or Proctocolitis or Rectocolitis or Ulcerative Rectocolitides or Ulcerative Rectocolitis or Ulcerative Proctocolitis or Proctosigmoiditis or Rectosigmoiditis or rectitis).tw,hw. | 763 |
| 14 | exp Phthirus/ or (Phthirus or Crab Louse? or Crab Lice? or pubic lice? or pubic louse? or Pediculus pubis or phthiriasis).tw,hw. | 667 |
| 15 | exp Scabies/ or (Scabies or Sarcoptic Mange or Sarcoptes scabiei).tw,hw. | 9455 |
| 16 | exp vaginitis/ or exp Vulvovaginitis/ or (vaginitis or colpitis or kolpitis or Vulvovaginitis or Vaginitides or Vulvovaginitides or Vulvovaginal Candidiasis or Vulvovaginal Moniliasis or Monilial Vaginitis or Genital Candidiasis or (vaginal adj3 yeast)).tw,hw. | 10867 |
| 17 | exp Urethritis/ or (urethritis or Urethritides).tw,hw. | 2142 |
| 18 | exp Human T-lymphotropic virus 1/ or exp Human T-cell leukemia virus 2/ or (HUMAN T-LYMPHOTROPIC VIRUS or HTLV or ATLV or Adult T-Cell Leukemia-Lymphoma Virus I or Human T-Cell Leukemia Virus I or Adult T Cell Leukemia Lymphoma Virus I or Human T Cell Leukemia Virus I or Human T lymphotropic virus 2 or Human T Lymphotropic Virus II or Human T-Lymphotropic Virus II or Human T Cell Leukemia Virus II or Human T lymphotropic virus 3 or STLV-3).tw,hw. | 8262 |
| 19 | (syphilis* or "great pox" or Chancre or Tabes Dorsalis or neurosyphilis or treponema pallidum or t pallidum or Chancroid* or lues or luetic disease or ulcus molle or Ducrey disease or "Ducrey's streptobacillosis" or ((ducrey or ducreyi) adj2 (Haemophilus or Hemophilus))).tw,hw. | 16604 |
| 20 | Epididymitis/ or epididymitis.tw,hw. | 1070 |
| 21 | Molluscum Contagiosum/ or (molluscum contagiosum or mollusca contagiosa or molluscum epitheliale).tw,hw. | 760 |
| 22 | or/1-21 [STBBIs] | 567951 |
| 23 | (transsexual* or transgender* or "trans gender*" or transpeople* or transm#n or transwom#n or nonbinary or "non binary" or "two spirit*" or "third gender*" or Transvestite or androgynous or "multi gender*" or "multigender*" or "gender nonconforming" or genderqueer or agender or "female to male" or FTM or "male to female" or MTF).tw,hw. | 14037 |
| 24 | ((trans adj3 (people or individual or individuals or person or persons or sexual* or man or men or male or female or youth* or woman or women or population* or gender* or sex worker* or adult* or patient* or participant* or masc* or femme* or feminine*)) or ((gender* or sex*) adj3 (fluid* or change or dysphoria or reversal or reassign* or transform* or transition*))).tw,hw. | 6818 |
| 25 | ((gender adj3 (neutral* or affirm* or inclusive or responsive or transformative)) or genderless or gender less).tw,hw. | 1603 |
| 26 | or/23-25 [Trans People] | 20804 |
| 27 | exp Canada/ or (canada* or canadia* or canadien* or ottawa* or british columbia* or colombie britannique* or vancouver* or alberta* or edmonton* or calgar* or saskatchewan* or regina* or saskatoon* or manitoba* or winnipeg* or ontari* or toronto* or quebec* or montreal* or new brunswick* or nouveau brunswick* or fredericton* or nova scotia* or nouvelle ecosse* or halifax* or haligonian* or prince edward island* or ile du prince edouard* or pei or charlottetown* or newfoundland* or terre neuve* or labrador* or nfld or yukon* or whitehorse* or northwest territor* or territoires du nord ouest* or nwt or yellowknife* or nunavut* or iqaluit*).tw,hw. | 241854 |
| 28 | 22 and 26 and 27 | 125 |
| 29 | limit 28 to yr="2013-Current" | 101 |

## PSYCINFO

Database(s): **APA PsycInfo**1806 to August Week 4 2023
Search Strategy:

| **#** | **Searches** | **Results** |
| --- | --- | --- |
| 1 | exp Sexually Transmitted Diseases/ | 51489 |
| 2 | ((("sexually transmitted" or Venereal) adj2 (disease* or infect*)) or STBBI? or blood borne infection? or STDs or STD or STI or STIs).tw,id. | 12551 |
| 3 | (hiv or hiv-1 or hiv-2 or hiv1 or hiv2 or hivaids or human immune deficiency virus or human immunodeficiency virus or human immuno-deficiency virus or human immune-deficiency virus or acquired immunodeficiency or acquired immune deficiency or acquired immuno-deficiency).tw,id. | 62002 |
| 4 | exp Herpes Simplex/ or (herpes or "hhv-1" or "hsv-1" or "hhv-2" or "hsv-2" or "herpesvirus" or herpes simplex virus or HSV or Kaposi Varicelliform Eruption or Herpetic Keratitis or Dendritic Keratitis or Herpetic Stomatitis or Herpetic Stomatitides or Eczema Vaccinatum or Eczema Herpeticum or Herpetic Keratitides or Dendritic Keratitides or Furrow Keratitis or Furrow Keratitides).tw,id. | 2625 |
| 5 | Human Papillomavirus/ or (((condyloma or condyla or condylatum or condylomata) adj3 (acuminatum or acuminata)) or verruca accuminata or vulvar condyloma or hpv or "human papillomavirus" or "Human Papilloma Virus" or ((genital or venereal or penile or perianal or anal or anogenital) adj3 wart*) or bowenoid papulosis or bowen dermatosis or bowenoid lesion or bowen disease?).tw,id. | 2565 |
| 6 | ("hep c" or hepatitis c or "hcv" or "hep b" or hepatitis b or "hbv" or "hep a" or hepatitis a or "hav").tw,id. | 4850 |
| 7 | (gonorrhea or gonococcus or gonorrhoeae or gonorrhoea or gonococci or gonococcal or gonococcosis).tw,id. | 886 |
| 8 | (chlamydi* or "c trachomatis" or C trachomatis or Chlamydiaceae* or Chlamydophila* or Lymphogranuloma Venereum or lymphopathia venerea or lymphopathia venereum or lymphogranuloma inguinale or inguinal lymphogranulomatosis or venereal lymphogranuloma or Durand-Nicholas-Favre disease or Favre-Durand disease or Frei disease or Nicholas Favre disease or Nicolas Favre disease or Nicolas-Durand-Favre disease).tw,id. | 1078 |
| 9 | (trichomoniasis or "trich" or Trichomoniases or Trichomonas).tw,id. | 191 |
| 10 | (Granuloma Inguinale or Granuloma Venereum or Donovanosis or granulomatis infection).tw,id. | 2 |
| 11 | (bacterial vaginosis or bacterial Vaginitides or Bacterial Vaginoses).tw,id. | 85 |
| 12 | (Adnexitis or Pelvic Inflammatory Disease* or Endometritis or Endomyometritis or Oophoritides or Oophoritis or Parametritis or Parametritides or Pelvic Cellulitides or Pelvic Cellulitis or Salpingitis or Salpingitides or pelvis abscess or pelvioperitonitis or metritis or Fitz-Hugh-Curtis syndrome).tw,id. | 113 |
| 13 | (Proctitis or Proctocolitis or Rectocolitis or Ulcerative Rectocolitides or Ulcerative Rectocolitis or Ulcerative Proctocolitis or Proctosigmoiditis or Rectosigmoiditis or rectitis).tw,id. | 29 |
| 14 | (Phthirus or Crab Louse? or Crab Lice? or pubic lice? or pubic louse? or Pediculus pubis or phthiriasis).tw,id. | 4 |
| 15 | (Scabies or Sarcoptic Mange or Sarcoptes scabiei).tw,id. | 41 |
| 16 | (vaginitis or colpitis or kolpitis or Vulvovaginitis or Vaginitides or Vulvovaginitides or Vulvovaginal Candidiasis or Vulvovaginal Moniliasis or Monilial Vaginitis or Genital Candidiasis or (vaginal adj3 yeast)).tw,id. | 76 |
| 17 | (urethritis or Urethritides).tw,id. | 55 |
| 18 | (HUMAN T-LYMPHOTROPIC VIRUS or HTLV or ATLV or Adult T-Cell Leukemia-Lymphoma Virus I or Human T-Cell Leukemia Virus I or Adult T Cell Leukemia Lymphoma Virus I or Human T Cell Leukemia Virus I or Human T lymphotropic virus 2 or Human T Lymphotropic Virus II or Human T-Lymphotropic Virus II or Human T Cell Leukemia Virus II or Human T lymphotropic virus 3 or STLV-3).tw,id. | 189 |
| 19 | (syphilis* or "great pox" or Chancre or Tabes Dorsalis or neurosyphilis or treponema pallidum or t pallidum or Chancroid* or lues or luetic disease or ulcus molle or Ducrey disease or "Ducrey's streptobacillosis" or ((ducrey or ducreyi) adj2 (Haemophilus or Hemophilus))).tw,id. | 2153 |
| 20 | epididymitis.tw,id. | 12 |
| 21 | (molluscum contagiosum or mollusca contagiosa or molluscum epitheliale).tw,id. | 5 |
| 22 | or/1-21 [STBBIs] | 78686 |
| 23 | Transgender/ or Transsexualism/ or Gender Reassignment/ or Gender Dysphoria/ or Gender Transition/ or Transvestism/ or Cisgender/ or Gender Nonbinary/ or Gender Nonconforming/ or Two-Spirit/ | 12542 |
| 24 | (transsexual* or transgender* or "trans gender*" or transpeople* or transm#n or transwom#n or nonbinary or "non binary" or "two spirit*" or "third gender*" or Transvestite or androgynous or "multi gender*" or "multigender*" or "gender nonconforming" or genderqueer or agender or "female to male" or FTM or "male to female" or MTF).tw,id. | 18407 |
| 25 | ((trans adj3 (people or individual or individuals or person or persons or sexual* or man or men or male or female or youth* or woman or women or population* or gender* or sex worker* or adult* or patient* or participant* or masc* or femme* or feminine*)) or ((gender* or sex*) adj3 (fluid* or change or dysphoria or reversal or reassign* or transform* or transition*))).tw,id. | 9558 |
| 26 | ((gender adj3 (neutral* or affirm* or inclusive or responsive or transformative)) or genderless or gender less).tw,id. | 3235 |
| 27 | or/23-26 [Trans] | 27757 |
| 28 | (canada* or canadia* or canadien* or ottawa* or british columbia* or colombie britannique* or vancouver* or alberta* or edmonton* or calgar* or saskatchewan* or regina* or saskatoon* or manitoba* or winnipeg* or ontari* or toronto* or quebec* or montreal* or new brunswick* or nouveau brunswick* or fredericton* or nova scotia* or nouvelle ecosse* or halifax* or haligonian* or prince edward island* or ile du prince edouard* or pei or charlottetown* or newfoundland* or terre neuve* or labrador* or nfld or yukon* or whitehorse* or northwest territor* or territoires du nord ouest* or nwt or yellowknife* or nunavut* or iqaluit*).tw,id. | 75478 |
| 29 | 22 and 27 and 28 | 76 |
| 30 | limit 29 to yr="2013-Current" | 57 |

## SCOPUS

(169 RESULTS)

( TITLE-ABS-KEY ( ( ( "sexually transmitted" OR venereal ) W/1 ( disease* OR infect* ) ) OR stbbi* OR "blood borne infection*" OR stds OR std OR sti OR stis ) OR TITLE-ABS-KEY ( hiv OR "hiv-1" OR "hiv-2" OR hiv1 OR hiv2 OR hivaids OR "human immune deficiency virus" OR "human immunodeficiency virus" OR "human immuno-deficiency virus" OR "human immune-deficiency virus" OR "acquired immunodeficiency" OR "acquired immune deficiency" OR "acquired immuno-deficiency" ) OR TITLE-ABS-KEY ( "herpesvirus" OR "herpes simplex virus" OR hsv OR "Kaposi Varicelliform Eruption" OR "Herpetic Keratitis" OR "Dendritic Keratitis" OR "Herpetic Stomatitis" OR "Herpetic Stomatitides" OR "Eczema Vaccinatum" OR "Eczema Herpeticum" OR "Herpetic Keratitides" OR "Dendritic Keratitides" OR "Furrow Keratitis" OR "Furrow Keratitides" ) OR TITLE-ABS-KEY ( ( ( condyloma OR condyla OR condylatum OR condylomata ) W/2 ( acuminatum OR acuminata ) ) OR "verruca accuminata" OR "vulvar condyloma" OR hpv OR "human papillomavirus" OR "Human Papilloma Virus" OR ( ( genital OR venereal OR penile OR perianal OR anal OR anogenital ) W/2 wart* ) OR "bowenoid papulosis" OR "bowen dermatosis" OR "bowenoid lesion" OR "bowen disease*" ) OR TITLE-ABS-KEY ( "hep c" OR "hepatitis c" OR "hcv" OR "hep b" OR "hepatitis b" OR "hbv" OR "hep a" OR "hepatitis a" OR "hav" ) OR TITLE-ABS-KEY ( gonorrhea OR gonococcus OR gonorrhoeae OR gonorrhoea OR gonococci OR gonococcal OR gonococcosis ) OR TITLE-ABS-KEY ( chlamydi* OR "c trachomatis" OR "C trachomatis" OR chlamydiaceae* OR chlamydophila* OR "Lymphogranuloma Venereum" OR "lymphopathia venerea" OR "lymphopathia venereum" OR "lymphogranuloma inguinale" OR "inguinal lymphogranulomatosis" OR "venereal lymphogranuloma" OR "Durand-Nicholas-Favre disease" OR "Favre-Durand disease" OR "Frei disease" OR "Nicholas Favre disease" OR "Nicolas Favre disease" OR "Nicolas-Durand-Favre disease" ) OR TITLE-ABS-KEY ( trichomoniasis OR "trich" OR trichomoniases OR trichomonas ) OR TITLE-ABS-KEY ( "Granuloma Inguinale" OR "Granuloma Venereum" OR donovanosis OR "granulomatis infection" ) OR TITLE-ABS-KEY ( "bacterial vaginosis" OR "bacterial Vaginitides" OR "Bacterial Vaginoses" ) OR TITLE-ABS-KEY ( adnexitis OR "Pelvic Inflammatory Disease*" OR endometritis OR endomyometritis OR oophoritides OR oophoritis OR parametritis OR parametritides OR "Pelvic Cellulitides" OR "Pelvic Cellulitis" OR salpingitis OR salpingitides OR "pelvis abscess" OR pelvioperitonitis OR metritis OR "Fitz-Hugh-Curtis syndrome" ) OR TITLE-ABS-KEY ( proctitis OR proctocolitis OR rectocolitis OR "Ulcerative Rectocolitides" OR "Ulcerative Rectocolitis" OR "Ulcerative Proctocolitis" OR proctosigmoiditis OR rectosigmoiditis OR rectitis ) OR TITLE-ABS-KEY ( phthirus OR "Crab Louse*" OR "Crab Lice*" OR "pubic lice*" OR "pubic louse*" OR "Pediculus pubis" OR phthiriasis ) OR TITLE-ABS-KEY ( scabies OR "Sarcoptic Mange" OR "Sarcoptes scabiei" ) OR TITLE-ABS-KEY ( vaginitis OR colpitis OR kolpitis OR vulvovaginitis OR vaginitides OR vulvovaginitides OR "Vulvovaginal Candidiasis" OR "Vulvovaginal Moniliasis" OR "Monilial Vaginitis" OR "Genital Candidiasis" OR ( vaginal W/2 yeast ) ) OR TITLE-ABS-KEY ( urethritis OR urethritides ) OR TITLE-ABS-KEY ( "HUMAN T-LYMPHOTROPIC VIRUS" OR htlv OR atlv OR "Adult T-Cell Leukemia-Lymphoma Virus I" OR "Human T-Cell Leukemia Virus I" OR "Adult T Cell Leukemia Lymphoma Virus I" OR "Human T Cell Leukemia Virus I" OR "Human T lymphotropic virus 2" OR "Human T Lymphotropic Virus II" OR "Human T-Lymphotropic Virus II" OR "Human T Cell Leukemia Virus II" OR "Human T lymphotropic virus 3" OR "STLV-3" ) OR TITLE-ABS-KEY ( syphilis* OR "great pox" OR chancre OR "Tabes Dorsalis" OR neurosyphilis OR "treponema pallidum" OR "t pallidum" OR chancroid* OR lues OR "luetic disease" OR "ulcus molle" OR "Ducrey disease" OR "Ducrey&apos;s streptobacillosis" OR ( ( ducrey OR ducreyi ) W/1 ( haemophilus OR hemophilus ) ) ) OR TITLE-ABS-KEY ( epididymitis OR "molluscum contagiosum" OR "mollusca contagiosa" OR "molluscum epitheliale" ) ) AND ( TITLE-ABS-KEY ( transsexual* OR transgender* OR "trans gender*" OR transpeople* OR transmen OR transwomen OR transman OR transwoman OR nonbinary OR "non binary" OR "two spirit*" OR "third gender*" OR transvestite OR androgynous OR "multi gender*" OR "multigender*" OR "gender nonconforming" OR genderqueer OR agender OR "female to male" OR ftm OR "male to female" OR mtf ) OR TITLE-ABS-KEY ( ( trans W/2 ( people OR individual OR individuals OR person OR persons OR sexual* OR man OR men OR male OR female OR youth* OR woman OR women OR population* OR gender* OR "sex worker*" OR adult* OR patient* OR participant* OR masc* OR femme* OR feminine* ) ) OR ( ( gender* OR sex* ) W/2 ( fluid* OR change OR dysphoria OR reversal OR reassign* OR transform* OR transition* ) ) ) OR TITLE-ABS-KEY ( ( gender W/2 ( neutral* OR affirm* OR inclusive OR responsive OR transformative ) ) OR genderless OR "gender less" ) ) AND TITLE-ABS-KEY ( canada* OR canadia* OR canadien* OR ottawa* OR "british columbia*" OR "colombie britannique*" OR vancouver* OR alberta* OR edmonton* OR calgar* OR saskatchewan* OR regina* OR saskatoon* OR manitoba* OR winnipeg* OR ontari* OR toronto* OR quebec* OR montreal* OR "new brunswick*" OR "nouveau brunswick*" OR fredericton* OR "nova scotia*" OR "nouvelle ecosse*" OR halifax* OR haligonian* OR "prince edward island*" OR "ile du prince edouard*" OR pei OR charlottetown* OR newfoundland* OR "terre neuve*" OR labrador* OR nfld OR yukon* OR whitehorse* OR "northwest territor*" OR "territoires du nord ouest*" OR nwt OR yellowknife* OR nunavut* OR iqaluit* ) AND PUBYEAR > 2012

**Grey literature search strategy – Government websites**

**Full search terms**

"sexually transmitted" OR STI OR HIV OR herpes OR “human papillomavirus virus” OR hepatitis OR gonorrhea OR gonorrhoea OR chlamydia OR trichomonas OR “granuloma inguinale” OR “bacterial vaginosis” OR “pelvic inflammatory disease” OR proctitis OR phthirus OR scabies OR vaginitis OR urethritis OR “human T-lymphotropic virus” OR syphilis OR epididymitis OR “molluscum contagiosum” OR “STBBI” OR “STBBIs” AND transgender OR trans

*Full search terms were too long to fit into the search box so were split into three sets, which were each used along with “AND transgender OR trans”*

**Set one**

transgender OR trans AND "sexually transmitted" OR STI OR STD OR HIV OR herpes OR hepatitis OR syphilis OR gonorrhea OR gonorrhoea OR chlamydia OR scabies OR vaginitis OR urethritis

**Set two**

transgender OR trans AND “human papillomavirus virus” OR trichomonas OR “granuloma inguinale” OR “bacterial vaginosis” OR “pelvic inflammatory disease” OR proctitis OR phthirus OR “human T-lymphotropic virus” OR epididymitis OR “molluscum contagiosum”

**Set three**

"STBBI" OR "STBBIs"

| Province or Territory | Public Health Department Website(s) |
| --- | --- |
| Alberta | www.albertahealthservices.ca |
| British Columbia | www.bccdc.ca |
| Manitoba | hwww.gov.mb.ca/health |
| Ontario | www.publichealthontario.ca |
| Saskatchewan | www.saskhealthauthority.ca/your-health |
| Quebec | www.inspq.qc.ca |
| Nova Scotia | www.nshealth.ca |
| New Brunswick | www2.gnb.ca |
| Newfoundland & Labrador | www.gov.nl.ca/hcs |
| PEI | www.princeedwardisland.ca/en/topic/public-health  www.princeedwardisland.ca/en/topic/health-and-wellness |
| Yukon | https://yukon.ca |
| Nunavut | www.gov.nu.ca/health |
| Northwest Territories | www.hss.gov.nt.ca  www.nthssa.ca |

Done using InPrivate window in Microsoft Edge Application (on [www.google.ca](http://www.google.ca))

**First set of search terms**

| Date | Organization Name | Pages flagged as relevant for checking |
| --- | --- | --- |
| Sept 26 | Alberta Health Services | None |
| Sept 29 | Public Health Ontario | 3 pages flagged – See below. |
| Sept 29 | BCCDC | 25 pages flagged - See below. |
| Sept 29 | PEI Department of Health and Wellness | None |
| Sept 29 | Nova Scotia Health Authority | None |
| Sept 29 | Nunavut Department of Health | None |
| Sept 29 | Newfoundland & Labrador Department of Health and Community Services | None |
| Oct 3 | Manitoba Health | 1 page flagged – See below. |
| Oct 6 | Quebec – INSPQ | 13 pages flagged - See below. |
| Oct 11 | Saskatchewan Health Authority | None |
| Oct 11 | Government of New Brunswick | None |
| Oct 12 | Northwest Territories Health & Social Services / Health & Social Services Authority | None |
| Oct 12 | Government of Yukon | None |

**Alberta**

site: www.albertahealthservices.ca AND (transgender OR trans) AND ("sexually transmitted" OR STI OR STD OR HIV OR herpes OR hepatitis OR syphilis OR gonorrhea OR gonorrhoea OR chlamydia OR scabies OR vaginitis OR urethritis)

Date: September 26, 2023

No relevant results found.

**Ontario**

site:www.publichealthontario.ca AND (transgender OR trans) AND ("sexually transmitted" OR STI OR STD OR HIV OR herpes OR hepatitis OR syphilis OR gonorrhea OR gonorrhoea OR chlamydia OR scabies OR vaginitis OR urethritis)

About 189 results

Done using InPrivate window in Microsoft Edge

Date: September 29, 2023

[Gonorrhea in Ontario: January 1, 2019 to December 31, 2021 (publichealthontario.ca)](https://www.publichealthontario.ca/-/media/Documents/G/2023/gonorrhea-ontario-epi-summary.ashx?rev=-1&la=fr)

[Infectious Syphilis in Ontario: January 1, 2019 to December 31, 2021 (publichealthontario.ca)](https://www.publichealthontario.ca/-/media/Documents/S/2023/syphillis-ontario-epi-summary.pdf?rev=3012dbbc382e41dda6510059bcf008d0&sc_lang=en)

[Chlamydia in Ontario: January 1, 2019 to December 31, 2021 (publichealthontario.ca)](https://www.publichealthontario.ca/-/media/Documents/C/2023/chlamydia-ontario-epi-summary.ashx?rev=-1&la=fr)

**British Columbia**

site:www.bccdc.ca AND (transgender OR trans) AND ("sexually transmitted" OR STI OR STD OR HIV OR herpes OR hepatitis OR syphilis OR gonorrhea OR gonorrhoea OR chlamydia OR scabies OR vaginitis OR urethritis)

About 494 results

Done using InPrivate window in Microsoft Edge

Date: September 29, 2023

[Syphilis_indicators_2023Q2.pdf (bccdc.ca)](http://www.bccdc.ca/resource-gallery/Documents/Statistics%20and%20Research/Statistics%20and%20Reports/STI/Syphilis_indicators_2023Q2.pdf)

[BC_syphilis_indicator_report_2022Q2_final.pdf (bccdc.ca)](http://www.bccdc.ca/resource-gallery/Documents/Statistics%20and%20Research/Statistics%20and%20Reports/STI/BC_syphilis_indicator_report_2022Q2_final.pdf)

[STI_Annual_Report_2017_final.pdf (bccdc.ca)](http://www.bccdc.ca/resource-gallery/Documents/Statistics%20and%20Research/Statistics%20and%20Reports/STI/STI_Annual_Report_2017_final.pdf)

[Syphilis_indicators_2023Q1.pdf (bccdc.ca)](http://www.bccdc.ca/resource-gallery/Documents/Statistics%20and%20Research/Statistics%20and%20Reports/STI/Syphilis_indicators_2023Q1.pdf)

[CPS_Report_STI_HIV_2010_annual_report_FINAL_20111122.pdf (bccdc.ca)](http://www.bccdc.ca/resource-gallery/Documents/Statistics%20and%20Research/Statistics%20and%20Reports/STI/CPS_Report_STI_HIV_2010_annual_report_FINAL_20111122.pdf)

[STI_Annual_Report_2011_20130327.pdf (bccdc.ca)](http://www.bccdc.ca/resource-gallery/Documents/Statistics%20and%20Research/Statistics%20and%20Reports/STI/STI_Annual_Report_2011_20130327.pdf)

[CPS Quarterly Surveillance Report - Q1 2023_FINAL.pdf (bccdc.ca)](http://www.bccdc.ca/resource-gallery/Documents/Statistics%20and%20Research/Statistics%20and%20Reports/STI/CPS%20Quarterly%20Surveillance%20Report%20-%20Q1%202023_FINAL.pdf)

[BC_syphilis_indicators_2021Q3_FINAL.pdf (bccdc.ca)](http://www.bccdc.ca/resource-gallery/Documents/Statistics%20and%20Research/Statistics%20and%20Reports/STI/BC_syphilis_indicators_2021Q3_FINAL.pdf)

[BCCDC_website_syphilis_indicators_2022Q4.pdf](http://www.bccdc.ca/resource-gallery/Documents/Statistics%20and%20Research/Statistics%20and%20Reports/STI/BCCDC_website_syphilis_indicators_2022Q4.pdf)

[STI_Annual_Report_2014-FINAL.pdf (bccdc.ca)](http://www.bccdc.ca/resource-gallery/Documents/Statistics%20and%20Research/Statistics%20and%20Reports/STI/STI_Annual_Report_2014-FINAL.pdf)

[STI_Annual_Report_2012FINAL.pdf (bccdc.ca)](http://www.bccdc.ca/resource-gallery/Documents/Statistics%20and%20Research/Statistics%20and%20Reports/STI/STI_Annual_Report_2012FINAL.pdf)

[STI_Annual_Report_2016.pdf (bccdc.ca)](http://www.bccdc.ca/resource-gallery/Documents/Statistics%20and%20Research/Statistics%20and%20Reports/STI/STI_Annual_Report_2016.pdf)

[STI_Annual_Report_2013FINAL.PDF (bccdc.ca)](http://www.bccdc.ca/resource-gallery/Documents/Statistics%20and%20Research/Statistics%20and%20Reports/STI/STI_Annual_Report_2013FINAL.PDF)

[STI_Annual_Report_2015-FINAL.pdf (bccdc.ca)](http://www.bccdc.ca/resource-gallery/Documents/Statistics%20and%20Research/Statistics%20and%20Reports/STI/STI_Annual_Report_2015-FINAL.pdf)

[CPS_HIV_Annual_Report_2012.pdf (bccdc.ca)](http://www.bccdc.ca/resource-gallery/Documents/Statistics%20and%20Research/Statistics%20and%20Reports/STI/CPS_HIV_Annual_Report_2012.pdf)

[2008AnnualSurveillancereport_Final_amended.pdf (bccdc.ca)](http://www.bccdc.ca/resource-gallery/Documents/Statistics%20and%20Research/Statistics%20and%20Reports/STI/2008AnnualSurveillancereport_Final_amended.pdf)

[HIV_Annual_Report_2011_20111011.pdf (bccdc.ca)](http://www.bccdc.ca/resource-gallery/Documents/Statistics%20and%20Research/Statistics%20and%20Reports/STI/HIV_Annual_Report_2011_20111011.pdf)

[hiv_annual_report_2014-final.pdf (bccdc.ca)](http://www.bccdc.ca/resource-gallery/documents/statistics%20and%20research/statistics%20and%20reports/sti/hiv_annual_report_2014-final.pdf)

[2001 (bccdc.ca)](http://www.bccdc.ca/resource-gallery/Documents/Statistics%20and%20Research/Statistics%20and%20Reports/STI/STI_HIVReport_HIVAIDSUpdate2001_20090520.pdf)

[HIV_Annual_Report_2013FINAL.PDF (bccdc.ca)](http://www.bccdc.ca/resource-gallery/Documents/Statistics%20and%20Research/Statistics%20and%20Reports/STI/HIV_Annual_Report_2013FINAL.PDF)

[2000.PDF (bccdc.ca)](http://www.bccdc.ca/resource-gallery/Documents/Statistics%20and%20Research/Statistics%20and%20Reports/STI/STI_HIVReport_HIVAIDSUpdate2000_20090520.pdf)

[CPS Monthly Surveillance Report - December 2022_FINAL.pdf (bccdc.ca)](http://www.bccdc.ca/resource-gallery/Documents/Statistics%20and%20Research/Statistics%20and%20Reports/STI/CPS%20Monthly%20Surveillance%20Report%20-%20December%202022_FINAL.pdf)

[CPS Monthly Surveillance Report - October 2022_FINAL.pdf (bccdc.ca)](http://www.bccdc.ca/resource-gallery/Documents/Statistics%20and%20Research/Statistics%20and%20Reports/STI/CPS%20Monthly%20Surveillance%20Report%20-%20October%202022_FINAL.pdf)

[CPS Monthly Surveillance Report - July 2021_FINAL.pdf (bccdc.ca)](http://www.bccdc.ca/resource-gallery/Documents/Statistics%20and%20Research/Statistics%20and%20Reports/STI/CPS%20Monthly%20Surveillance%20Report%20-%20July%202021_FINAL.pdf)

[End.PDF (bccdc.ca)](http://www.bccdc.ca/resource-gallery/Documents/Statistics%20and%20Research/Statistics%20and%20Reports/STI/STI_HIVReport_HIVAIDSUpdate1999_20090520.pdf)

**Newfoundland & Labrador**

site:www.gov.nl.ca/hcs/publichealth AND (transgender OR trans) AND ("sexually transmitted" OR STI OR STD OR HIV OR herpes OR hepatitis OR syphilis OR gonorrhea OR gonorrhoea OR chlamydia OR scabies OR vaginitis OR urethritis)

0 RESULTS WITH SET #1

Done using InPrivate window in Microsoft Edge

Date: September 29, 2023

--------

site:www.gov.nl.ca/hcs AND (transgender OR trans) AND ("sexually transmitted" OR STI OR STD OR HIV OR herpes OR hepatitis OR syphilis OR gonorrhea OR gonorrhoea OR chlamydia OR scabies OR vaginitis OR urethritis)

About 157 results 🡨 BUT there were only 32 records to scan

Done using InPrivate window in Microsoft Edge

Date: September 29, 2023

No relevant results found.

**Nova Scotia**

site:www.nshealth.ca AND (transgender OR trans) AND ("sexually transmitted" OR STI OR STD OR HIV OR herpes OR hepatitis OR syphilis OR gonorrhea OR gonorrhoea OR chlamydia OR scabies OR vaginitis OR urethritis)

About 59 results 🡨 BUT there were only 37 results available

Done using InPrivate window in Microsoft Edge

Date: September 29, 2023

No relevant results found.

**Nunavut**

site:www.gov.nu.ca/health AND (transgender OR trans) AND ("sexually transmitted" OR STI OR STD OR HIV OR herpes OR hepatitis OR syphilis OR gonorrhea OR gonorrhoea OR chlamydia OR scabies OR vaginitis OR urethritis)

0 RESULTS WITH SET #1

Done using InPrivate window in Microsoft Edge

Date: September 29, 2023

**Prince Edward Island**

site:www.princeedwardisland.ca/en/topic/public-health AND (transgender OR trans) AND ("sexually transmitted" OR STI OR STD OR HIV OR herpes OR hepatitis OR syphilis OR gonorrhea OR gonorrhoea OR chlamydia OR scabies OR vaginitis OR urethritis)

0 RESULTS WITH SET #1

Done using InPrivate window in Microsoft Edge

Date: September 29, 2023

----------

site:www.princeedwardisland.ca/en/topic/health-and-wellness AND (transgender OR trans) AND ("sexually transmitted" OR STI OR STD OR HIV OR herpes OR hepatitis OR syphilis OR gonorrhea OR gonorrhoea OR chlamydia OR scabies OR vaginitis OR urethritis)

0 RESULTS WITH SET #1

Done using InPrivate window in Microsoft Edge

Date: September 29, 2023

**Manitoba**

site:www.gov.mb.ca/health AND (transgender OR trans) AND ("sexually transmitted" OR STI OR STD OR HIV OR herpes OR hepatitis OR syphilis OR gonorrhea OR gonorrhoea OR chlamydia OR scabies OR vaginitis OR urethritis)

45 RESULTS WITH SET #1

Done using InPrivate window in Microsoft Edge

Date: October 3, 2023

[dec2016.pdf (gov.mb.ca)](https://www.gov.mb.ca/health/publichealth/surveillance/hivaids/docs/dec2016.pdf)

**Quebec**

site:www.inspq.qc.ca AND (transgenre OR trans) AND ("transmissible sexuellement" OR ITS OR MST OR VIH OR herpès OR hépatite OR syphilis OR gonorrhée OR gonorrhoeae OR chlamydia OR gale OR vaginite OR urétrite)

Environ 664 résultats

Done using InPrivate window in Microsoft Edge

Date: October 6, 2023

[Programme de surveillance de l'infection par le virus de l'immunodéficience humaine (VIH) au Québec : rapport 2019 (inspq.qc.ca)](https://www.inspq.qc.ca/sites/default/files/publications/2706_programme_surveillance_infection_2019_0.pdf)

[Programme de surveillance de l’infection par le virus de l’immunodéficience humaine (VIH) au Québec : rapport annuel 2021 (inspq.qc.ca)](https://www.inspq.qc.ca/sites/default/files/2023-07/3361-programme-surveillance-vih-2021.pdf)

[Programme de surveillance de l'infection par le virus de l'immunodéficience humaine (VIH) au Québec - Rapport 2020 (inspq.qc.ca)](https://www.inspq.qc.ca/sites/default/files/publications/2889-programme-surveillance-vih-2020.pdf)

[Stratégies de distribution des autotests du VIH : comment joindre les populations prioritaires? (inspq.qc.ca)](https://www.inspq.qc.ca/sites/default/files/publications/3318-strategies-distribution-autotest_vih.pdf)

[Portrait des infections transmissibles sexuellement et par le sang (ITSS) au Québec : année 2017 et projection 2018 (inspq.qc.ca)](https://www.inspq.qc.ca/sites/default/files/publications/2471_infections_transmissibles_sexuellement_sang_2017.pdf)

[Portrait des infections transmissibles sexuellement et par le sang (ITSS) au QUébec (inspq.qc.ca)](https://www.inspq.qc.ca/sites/default/files/publications/2783-portrait-infections-transmissibles-sexuellement-sang.pdf)

[VIH et hépatite C en prison (inspq.qc.ca)](https://www.inspq.qc.ca/sites/default/files/documents/formation/itss/reseau_juridique_13_feuillets.pdf)

[Portrait des infections transimissibles sexuellement et par le sang (ITSS) au Québec - Anneée 2018 et Projection 2019 (inspq.qc.ca)](https://www.inspq.qc.ca/sites/default/files/publications/2612_infections_transmissibles_sexuellement_sang.pdf)

[Surveillance of Gonococcal Infection Treatment Failures 2015-2018 in Quebec, Canada (inspq.qc.ca)](https://www.inspq.qc.ca/sites/default/files/documents/itss/19-328-02a_affiche_scientifique_gonococcal_treatment_failure_vf_br.pdf)

[Optimiser le dépistage du virus de l'immunodficience humaine au Québec à l'ère des nouvelles stratégies de prévention (inspq.qc.ca)](https://www.inspq.qc.ca/sites/default/files/publications/2489_optmisier_depistage_vih.pdf)

[JASP 2017 (inspq.qc.ca)](https://www.inspq.qc.ca/sites/default/files/jasp/archives/2017/jasp2017_harsahitss_riyasfadel_2.pdf)

[Le mois des Fiertés au Québec | Institut national de santé publique du Québec (inspq.qc.ca)](https://www.inspq.qc.ca/es/node/32650?themekey-theme=mobile&page=8)

[Microsoft PowerPoint - JASP 202017_JOtis_06-12-2017.pptx (inspq.qc.ca)](https://www.inspq.qc.ca/sites/default/files/jasp/archives/2017/jasp2017_harsahitss_joanneotis.pdf)

[Sources | Institut national de santé publique du Québec (inspq.qc.ca)](https://www.inspq.qc.ca/intimidation/sources)

**Saskatchewan**

site:www.saskhealthauthority.ca/your-health AND (transgender OR trans) AND ("sexually transmitted" OR STI OR STD OR HIV OR herpes OR hepatitis OR syphilis OR gonorrhea OR gonorrhoea OR chlamydia OR scabies OR vaginitis OR urethritis)

About 777 results

Done using InPrivate window in Microsoft Edge

Date: October 11, 2023

No relevant results

**New Brunswick**

site:www2.gnb.ca AND (transgender OR trans) AND ("sexually transmitted" OR STI OR STD OR HIV OR herpes OR hepatitis OR syphilis OR gonorrhea OR gonorrhoea OR chlamydia OR scabies OR vaginitis OR urethritis)

About 124 results

Done using InPrivate window in Microsoft Edge

Date: October 11, 2023

No relevant results.

**Yukon**

site:https://yukon.ca AND (transgender OR trans) AND ("sexually transmitted" OR STI OR STD OR HIV OR herpes OR hepatitis OR syphilis OR gonorrhea OR gonorrhoea OR chlamydia OR scabies OR vaginitis OR urethritis)

About 129 results

Done using InPrivate window in Microsoft Edge

Date: October 12, 2023

No relevant results

**Northwest Territories**

site:www.hss.gov.nt.ca AND (transgender OR trans) AND ("sexually transmitted" OR STI OR STD OR HIV OR herpes OR hepatitis OR syphilis OR gonorrhea OR gonorrhoea OR chlamydia OR scabies OR vaginitis OR urethritis)

About 112 results <- only 14 results visible

Done using InPrivate window in Microsoft Edge

Date: October 12, 2023

No relevant results

------

site:www.nthssa.ca AND (transgender OR trans) AND ("sexually transmitted" OR STI OR STD OR HIV OR herpes OR hepatitis OR syphilis OR gonorrhea OR gonorrhoea OR chlamydia OR scabies OR vaginitis OR urethritis)

0 RESULTS

Done using InPrivate window in Microsoft Edge

Date: October 12, 2023

**Second set of search terms**

| Date | Organization Name | Pages flagged as relevant for checking |
| --- | --- | --- |
| Oct 4 | Alberta Health Services | None |
| Oct 3 | Public Health Ontario | None |
| Oct 3 | BCCDC | [1](http://www.bccdc.ca/resource-gallery/Documents/Statistics%20and%20Research/Statistics%20and%20Reports/STI/STI_Annual_STIAnnualReport1999_20090520.pdf) page flagged – See below. |
| Oct 4 | PEI Department of Health and Wellness | None |
| Oct 4 | Nova Scotia Health Authority | None |
| Oct 4 | Nunavut Department of Health | None |
| Oct 4 | Newfoundland & Labrador Department of Health and Community Services | None |
| Oct 4 | Manitoba Health | None |
| Oct 6 | Quebec – INSPQ | 4 pages flagged – See below. |
| Oct 12 | Saskatchewan Health Authority | None |
| Oct 11 | Government of New Brunswick | None |
| Oct 12 | Northwest Territories Health & Social Services / Health & Social Services Authority | None |
| Oct 11 | Government of Yukon | None |

**ONTARIO**

site:www.publichealthontario.ca AND (transgender OR trans) AND (“human papillomavirus virus” OR trichomonas OR “granuloma inguinale” OR “bacterial vaginosis” OR “pelvic inflammatory disease” OR proctitis OR phthirus OR “human T-lymphotropic virus” OR epididymitis OR “molluscum contagiosum”)

About 6 results

Done using InPrivate window in Microsoft Edge

Date: October 3, 2023

No relevant results

**BRITISH COLUMBIA**

site:www.bccdc.ca AND (transgender OR trans) AND (“human papillomavirus virus” OR trichomonas OR “granuloma inguinale” OR “bacterial vaginosis” OR “pelvic inflammatory disease” OR proctitis OR phthirus OR “human T-lymphotropic virus” OR epididymitis OR “molluscum contagiosum”)

About 58 results

Done using InPrivate window in Microsoft Edge

Date: October 3, 2023

NEW: [STD 1999 Annual Report (bccdc.ca)](http://www.bccdc.ca/resource-gallery/Documents/Statistics%20and%20Research/Statistics%20and%20Reports/STI/STI_Annual_STIAnnualReport1999_20090520.pdf)

**the following studies were found in set #1 search as well**

[STI_Annual_Report_2017_final.pdf (bccdc.ca)](http://www.bccdc.ca/resource-gallery/Documents/Statistics%20and%20Research/Statistics%20and%20Reports/STI/STI_Annual_Report_2017_final.pdf)

[STI_Annual_Report_2016.pdf (bccdc.ca)](http://www.bccdc.ca/resource-gallery/Documents/Statistics%20and%20Research/Statistics%20and%20Reports/STI/STI_Annual_Report_2016.pdf)

[STI_Annual_Report_2011_20130327.pdf (bccdc.ca)](http://www.bccdc.ca/resource-gallery/Documents/Statistics%20and%20Research/Statistics%20and%20Reports/STI/STI_Annual_Report_2011_20130327.pdf)

[CPS_Report_STI_HIV_2010_annual_report_FINAL_20111122.pdf (bccdc.ca)](http://www.bccdc.ca/resource-gallery/Documents/Statistics%20and%20Research/Statistics%20and%20Reports/STI/CPS_Report_STI_HIV_2010_annual_report_FINAL_20111122.pdf)

[STI_Annual_Report_2012FINAL.pdf (bccdc.ca)](http://www.bccdc.ca/resource-gallery/Documents/Statistics%20and%20Research/Statistics%20and%20Reports/STI/STI_Annual_Report_2012FINAL.pdf)

[STI_Annual_Report_2015-FINAL.pdf (bccdc.ca)](http://www.bccdc.ca/resource-gallery/Documents/Statistics%20and%20Research/Statistics%20and%20Reports/STI/STI_Annual_Report_2015-FINAL.pdf)

[STI_Annual_Report_2013FINAL.PDF (bccdc.ca)](http://www.bccdc.ca/resource-gallery/Documents/Statistics%20and%20Research/Statistics%20and%20Reports/STI/STI_Annual_Report_2013FINAL.PDF)

[STI_Annual_Report_2014-FINAL.pdf (bccdc.ca)](http://www.bccdc.ca/resource-gallery/Documents/Statistics%20and%20Research/Statistics%20and%20Reports/STI/STI_Annual_Report_2014-FINAL.pdf)

[2008AnnualSurveillancereport_Final_amended.pdf (bccdc.ca)](http://www.bccdc.ca/resource-gallery/Documents/Statistics%20and%20Research/Statistics%20and%20Reports/STI/2008AnnualSurveillancereport_Final_amended.pdf)

**NEWFOUNDLAND & LABRADOR**

site:www.gov.nl.ca/hcs/publichealth AND (transgender OR trans) AND (“human papillomavirus virus” OR trichomonas OR “granuloma inguinale” OR “bacterial vaginosis” OR “pelvic inflammatory disease” OR proctitis OR phthirus OR “human T-lymphotropic virus” OR epididymitis OR “molluscum contagiosum”)

0 RESULTS WITH SET #2

Done using InPrivate window in Microsoft Edge

Date: October 4, 2023

-------

site:www.gov.nl.ca/hcs AND (transgender OR trans) AND (“human papillomavirus virus” OR trichomonas OR “granuloma inguinale” OR “bacterial vaginosis” OR “pelvic inflammatory disease” OR proctitis OR phthirus OR “human T-lymphotropic virus” OR epididymitis OR “molluscum contagiosum”)

About 2 results

Done using InPrivate window in Microsoft Edge

Date: October 4, 2023

No relevant results

**Nova Scotia**

site:www.nshealth.ca AND (transgender OR trans) AND (“human papillomavirus virus” OR trichomonas OR “granuloma inguinale” OR “bacterial vaginosis” OR “pelvic inflammatory disease” OR proctitis OR phthirus OR “human T-lymphotropic virus” OR epididymitis OR “molluscum contagiosum”)

About 1 result

Done using InPrivate window in Microsoft Edge

Date: October 4, 2023

No relevant results

**Nunavut**

site:www.gov.nu.ca/health AND (transgender OR trans) AND (“human papillomavirus virus” OR trichomonas OR “granuloma inguinale” OR “bacterial vaginosis” OR “pelvic inflammatory disease” OR proctitis OR phthirus OR “human T-lymphotropic virus” OR epididymitis OR “molluscum contagiosum”)

0 RESULTS WITH SET #2

Done using InPrivate window in Microsoft Edge

Date: October 4, 2023

**Prince Edward Island**

site:www.princeedwardisland.ca/en/topic/public-health AND (transgender OR trans) AND (“human papillomavirus virus” OR trichomonas OR “granuloma inguinale” OR “bacterial vaginosis” OR “pelvic inflammatory disease” OR proctitis OR phthirus OR “human T-lymphotropic virus” OR epididymitis OR “molluscum contagiosum”)

0 RESULTS WITH SET #2

Done using InPrivate window in Microsoft Edge

Date: October 4, 2023

----------

site:www.princeedwardisland.ca/en/topic/health-and-wellness AND (transgender OR trans) AND (“human papillomavirus virus” OR trichomonas OR “granuloma inguinale” OR “bacterial vaginosis” OR “pelvic inflammatory disease” OR proctitis OR phthirus OR “human T-lymphotropic virus” OR epididymitis OR “molluscum contagiosum”)

0 RESULTS WITH SET #2

Done using InPrivate window in Microsoft Edge

Date: October 4, 2023

**Manitoba**

site:www.gov.mb.ca/health AND (transgender OR trans) AND (“human papillomavirus virus” OR trichomonas OR “granuloma inguinale” OR “bacterial vaginosis” OR “pelvic inflammatory disease” OR proctitis OR phthirus OR “human T-lymphotropic virus” OR epididymitis OR “molluscum contagiosum”)

About 7 results

Done using InPrivate window in Microsoft Edge

Date: October 4, 2023

No relevant results

**Alberta**

site:www.albertahealthservices.ca AND (transgender OR trans) AND (“human papillomavirus virus” OR trichomonas OR “granuloma inguinale” OR “bacterial vaginosis” OR “pelvic inflammatory disease” OR proctitis OR phthirus OR “human T-lymphotropic virus” OR epididymitis OR “molluscum contagiosum”)

About 3 results

Done using InPrivate window in Microsoft Edge

Date: October 4, 2023

No relevant results

**Quebec**

site:www.inspq.qc.ca AND (transgenre OR trans) AND (“virus du papillome humain” OR trichomonas OR “granulome inguinal” OR “vaginose bactérienne” OR “maladie inflammatoire pelvienne” OR rectite OR phthirus OR “virus T-lymphotrope humain” OR épididymite OR “molluscum contagiosum”)

Environ 80 résultats

Done using InPrivate window in Microsoft Edge

Date: October 6, 2023

[Étude PIXEL – Portrait de la santé sexuelle des jeunes adultes au Québec, 2013-2014 (inspq.qc.ca)](https://www.inspq.qc.ca/sites/default/files/publications/2138_etude_pixel_rapport_methodologique.pdf)

[communications_affichees_2017.pdf (inspq.qc.ca)](https://www.inspq.qc.ca/sites/default/files/jasp/archives/2017/communications_affichees_2017.pdf)

[Rapport intégré : épidémiologie des infections transmissibles sexuellement et par le sang au Québec (inspq.qc.ca)](https://www.inspq.qc.ca/sites/default/files/publications/1522_rappintegreepidemioitssqc.pdf)

[Portrait des infections transmissibles sexuellement et par le sang (ITSS) au Québec : année 2021 et projections 2022 (inspq.qc.ca)](https://www.inspq.qc.ca/sites/default/files/2023-07/3364-portrait-itss-2021-projections-2022.pdf)

site:www.inspq.qc.ca AND (transgenre OR trans) AND (“proctite” OR “lymphotropique”)

Environ 1 résultats

Done using InPrivate window in Microsoft Edge

Date: October 6, 2023

No relevant results

**Saskatchewan**

site:www.saskhealthauthority.ca/your-health AND (transgender OR trans) AND (“human papillomavirus virus” OR trichomonas OR “granuloma inguinale” OR “bacterial vaginosis” OR “pelvic inflammatory disease” OR proctitis OR phthirus OR “human T-lymphotropic virus” OR epididymitis OR “molluscum contagiosum”)

About 202 results

Done using InPrivate window in Microsoft Edge

Date: October 12, 2023

No relevant results

**New Brunswick**

site:www2.gnb.ca AND (transgender OR trans) AND (“human papillomavirus virus” OR trichomonas OR “granuloma inguinale” OR “bacterial vaginosis” OR “pelvic inflammatory disease” OR proctitis OR phthirus OR “human T-lymphotropic virus” OR epididymitis OR “molluscum contagiosum”)

About 1 results

Done using InPrivate window in Microsoft Edge

Date: October 11, 2023

No relevant results.

**Yukon**

site:https://yukon.ca AND (transgender OR trans) AND (“human papillomavirus virus” OR trichomonas OR “granuloma inguinale” OR “bacterial vaginosis” OR “pelvic inflammatory disease” OR proctitis OR phthirus OR “human T-lymphotropic virus” OR epididymitis OR “molluscum contagiosum”)

About 1 results

Done using InPrivate window in Microsoft Edge

Date: October 11, 2023

No relevant results

**Northwest Territories**

site:www.hss.gov.nt.ca AND (transgender OR trans) AND (“human papillomavirus virus” OR trichomonas OR “granuloma inguinale” OR “bacterial vaginosis” OR “pelvic inflammatory disease” OR proctitis OR phthirus OR “human T-lymphotropic virus” OR epididymitis OR “molluscum contagiosum”)

0 RESULTS

Done using InPrivate window in Microsoft Edge

Date: October 12, 2023

----------

site:www.nthssa.ca AND (transgender OR trans) AND (“human papillomavirus virus” OR trichomonas OR “granuloma inguinale” OR “bacterial vaginosis” OR “pelvic inflammatory disease” OR proctitis OR phthirus OR “human T-lymphotropic virus” OR epididymitis OR “molluscum contagiosum”)

0 RESULTS

Done using InPrivate window in Microsoft Edge

Date: October 12, 2023

**Third set of search terms**

| Date | Organization Name | Specific pages flagged as relevant for checking |
| --- | --- | --- |
| Oct 4 | Alberta Health Services | None |
| Oct 4 | Public Health Ontario | None |
| Oct 4 | BCCDC | None |
| Oct 4 | PEI Department of Health and Wellness | None |
| Oct 4 | Nova Scotia Health Authority | None |
| Oct 4 | Nunavut Department of Health | None |
| Oct 4 | Newfoundland & Labrador Department of Health and Community Services | None |
| Oct 4 | Manitoba Health | None |
| Oct 10 | Quebec – INSPQ | 1 page flagged – See below. |
| Oct 12 | Saskatchewan Health Authority | None |
| Oct 12 | Government of New Brunswick | None |
| Oct 12 | Northwest Territories Health & Social Services / Health & Social Services Authority | None |
| Oct 12 | Government of Yukon | None |

**Ontario**

site:www.publichealthontario.ca AND (transgender OR trans) AND ("STBBI" OR "STBBIs")

10 results

Done using InPrivate window in Microsoft Edge

Date: October 4, 2023

No relevant results

**British Columbia**

site:www.bccdc.ca AND (transgender OR trans) AND ("STBBI" OR "STBBIs")

About 8 results

Done using InPrivate window in Microsoft Edge

Date: October 4, 2023

No relevant results

**Newfoundland & Labrador**

site:www.gov.nl.ca/hcs/publichealth AND (transgender OR trans) AND ("STBBI" OR "STBBIs")

0 RESULTS

Done using InPrivate window in Microsoft Edge

Date: October 4, 2023

-------

site:www.gov.nl.ca/hcs AND (transgender OR trans) AND ("STBBI" OR "STBBIs")

About 2 results

Done using InPrivate window in Microsoft Edge

Date: October 4, 2023

No relevant results

**Nova Scotia**

site:www.nshealth.ca AND (transgender OR trans) AND ("STBBI" OR "STBBIs")

About 6 results

Done using InPrivate window in Microsoft Edge

Date: October 4, 2023

No relevant results

**Nunavut**

site:www.gov.nu.ca/health AND (transgender OR trans) AND ("STBBI" OR "STBBIs")

0 Results

Done using InPrivate window in Microsoft Edge

Date: October 4, 2023

**Alberta**

site:www.albertahealthservices.ca AND (transgender OR trans) AND ("STBBI" OR "STBBIs")

0 Results

Done using InPrivate window in Microsoft Edge

Date: May 6, 2024

**Prince Edward Island**

site:www.princeedwardisland.ca/en/topic/public-health AND ("STBBI" OR "STBBIs")

0 results

Done using InPrivate window in Microsoft Edge

Date: October 4, 2023

----------

site:www.princeedwardisland.ca/en/topic/health-and-wellness AND ("STBBI" OR "STBBIs")

0 results

Done using InPrivate window in Microsoft Edge

Date: October 4, 2023

**Manitoba**

site:www.gov.mb.ca/health AND (transgender OR trans) AND ("STBBI" OR "STBBIs")

About 46 results

Done using InPrivate window in Microsoft Edge

Date: October 4, 2023

No relevant results

**Quebec**

site:www.inspq.qc.ca AND (transgenre OR trans) AND ("ITSS")

Done using InPrivate window in Microsoft Edge

Environ 580 résultats

Date: October 10, 2023

[L'intimidation vécue par les jeunes de la diversité sexuelle ou de genre | Institut national de santé publique du Québec (inspq.qc.ca)](https://www.inspq.qc.ca/intimidation/personnes-de-la-diversite-sexuelle-ou-de-genre/jeunes)

**Saskatchewan**

site:www.saskhealthauthority.ca/your-health AND (transgender OR trans) AND ("STBBI" OR "STBBIs")

0 RESULTS

Done using InPrivate window in Microsoft Edge

Date: October 12, 2023

**New Brunswick**

site:www2.gnb.ca AND (transgender OR trans) AND ("STBBI" OR "STBBIs")

About 6 results

Done using InPrivate window in Microsoft Edge

Date: October 12, 2023

No relevant results

**Yukon**

site:https://yukon.ca AND (transgender OR trans) AND ("STBBI" OR "STBBIs")

0 RESULTS

Done using InPrivate window in Microsoft Edge

Date: October 12, 2023

**Northwest Territories**

site:www.hss.gov.nt.ca AND (transgender OR trans) AND ("STBBI" OR "STBBIs")

About 2 results

Done using InPrivate window in Microsoft Edge

Date: October 12, 2023

No relevant results

**----------**

site:www.nthssa.ca AND (transgender OR trans) AND ("STBBI" OR "STBBIs")

0 RESULTS

Done using InPrivate window in Microsoft Edge

Date: October 12, 2023

**Grey literature search strategy – Organization websites**

**Set one**

transgender OR trans AND "sexually transmitted" OR STI OR STD OR HIV OR herpes OR hepatitis OR syphilis OR gonorrhea OR gonorrhoea OR chlamydia OR scabies OR vaginitis OR urethritis

**Set two**

transgender OR trans AND “human papillomavirus virus” OR trichomonas OR “granuloma inguinale” OR “bacterial vaginosis” OR “pelvic inflammatory disease” OR proctitis OR phthirus OR “human T-lymphotropic virus” OR epididymitis OR “molluscum contagiosum”

**All together, for reference**

"sexually transmitted" OR STI OR HIV OR herpes OR “human papillomavirus virus” OR hepatitis OR gonorrhea OR gonorrhoea OR chlamydia OR trichomonas OR “granuloma inguinale” OR “bacterial vaginosis” OR “pelvic inflammatory disease” OR proctitis OR phthirus OR scabies OR vaginitis OR urethritis OR “human T-lymphotropic virus” OR syphilis OR epididymitis OR “molluscum contagiosum” AND transgender OR trans

| Organization | Organization Website |
| --- | --- |
| REACH Nexus | [www.reachnexus.ca](https://www.reachnexus.ca) |
| Ontario HIV Treatment Network | [www.ohtn.on.ca](http://www.ohtn.on.ca) |
| Catie | [www.catie.ca](http://www.catie.ca) |
| Action Canada SHR | [www.actioncanadashr.org](http://www.actioncanadashr.org) |
| CBRC | [www.cbrc.net](http://www.cbrc.net) |
| Gay Mens Sexual Health Alliance | [www.gmsh.ca](http://www.gmsh.ca) |
| BC Hepatitis C Network | [www.bchep.org](http://www.bchep.org) |
| The Aboriginal HIV/AIDS Community-Based Research Collaborative Centre (AHA Centre) | [www.caan.ca](http://www.caan.ca) |

Done using InPrivate window in Microsoft Edge Application (on [www.google.ca](http://www.google.ca))

**First set of search terms**

| Date | Organization Name | Specific pages flagged as relevant for checking |
| --- | --- | --- |
| Oct 5 | REACH Nexus | No relevant results |
| Oct 5 | Ontario HIV Treatment Network | 16 pages flagged - See below. |
| Oct 5 | Catie | 4 pages flagged - See below. |
| Oct 6 | Action Canada SHR | 4 pages flagged - See below. |
| Oct 6 | CBRC | 6 pages flagged - See below. |
| Oct 10 | Gay Mens Sexual Health Alliance | 1 page flagged – See below. |
| Oct 10 | BC Hepatitis C Network | No relevant results |
| Oct 10 | Canadian Aboriginal AIDS Network (CAAN) | 9 pages flagged - See below |

**SET #1**

**REACH Nexus**

site:www.reachnexus.ca AND (transgender OR trans) AND ("sexually transmitted" OR STI OR STD OR HIV OR herpes OR hepatitis OR syphilis OR gonorrhea OR gonorrhoea OR chlamydia OR scabies OR vaginitis OR urethritis)

10 results

Done using InPrivate window in Microsoft Edge

Date: October 5, 2023

No relevant results

**Ontario HIV Treatment Network**

site:www.ohtn.on.ca AND (transgender OR trans) AND ("sexually transmitted" OR STI OR STD OR HIV OR herpes OR hepatitis OR syphilis OR gonorrhea OR gonorrhoea OR chlamydia OR scabies OR vaginitis OR urethritis)

About 1,040 results

Done using InPrivate window in Microsoft Edge

Date: October 5, 2023

[Barriers to accessing health care among transgender individuals – The Ontario HIV Treatment Network (ohtn.on.ca)](https://www.ohtn.on.ca/rapid-response-barriers-to-accessing-health-care-among-transgender-individuals/)

[Programs to improve the sexual health and well-being of transgender individuals – The Ontario HIV Treatment Network (ohtn.on.ca)](https://www.ohtn.on.ca/rapid-response-104-programs-to-improve-the-sexual-health-and-well-being-of-transgender-individuals-2/)

[Awareness, accessibility and uptake of pre-exposure prophylaxis (PrEP) among cisgender and transgender women at risk of HIV infection – The Ontario HIV Treatment Network (ohtn.on.ca)](https://www.ohtn.on.ca/rapid-response-awareness-accessibility-and-uptake-of-pre-exposure-prophylaxis-among-cisgender-and-transgender-women-at-risk-of-hiv-infection/)

[RR33.TransmenandHIV.FINAL.pub (ohtn.on.ca)](https://www.ohtn.on.ca/Pages/Knowledge-Exchange/Rapid-Responses/Documents/RR33-2010-TransMen-HIV.pdf)

[A review of internet-based testing services for HIV and sexually transmitted infections (STIs) – The Ontario HIV Treatment Network (ohtn.on.ca)](https://www.ohtn.on.ca/rapid-response-a-review-of-internet-based-testing-services-for-hiv-and-sexually-transmitted-infections-stis/)

[OHTN-Evidence-Report-IDAHOBIT.pdf](https://www.ohtn.on.ca/wp-content/uploads/2023/06/OHTN-Evidence-Report-IDAHOBIT.pdf)

[Targeted interventions for men who have sex with men, trans women, and people who inject drugs – The Ontario HIV Treatment Network (ohtn.on.ca)](https://www.ohtn.on.ca/targeted-interventions-for-men-who-have-sex-with-men-trans-women-and-people-who-inject-drugs/)

[Indigenous-Report-Overview.pdf (ohtn.on.ca)](https://www.ohtn.on.ca/wp-content/uploads/2015/10/Indigenous-Report-Overview.pdf)

[Intersectionality in HIV and other health-related research – The Ontario HIV Treatment Network (ohtn.on.ca)](https://www.ohtn.on.ca/rapid-response-68-intersectionality-in-hiv-and-other-health-related-research/)

[HIV-related stigma in relation to health care professionals in Canada – The Ontario HIV Treatment Network (ohtn.on.ca)](https://www.ohtn.on.ca/rapid-response-94-hiv-related-stigma-in-relation-to-health-care-professionals-in-canada-2/)

[Facilitators and barriers to health care for lesbian, gay and bisexual people – The Ontario HIV Treatment Network (ohtn.on.ca)](https://www.ohtn.on.ca/rapid-response-79-facilitators-and-barriers-to-health-care-for-lesbian-gay-and-bisexual-lgb-people/)

[The impact of location on implementation of HIV/STI prevention interventions among LGBTQ communities – The Ontario HIV Treatment Network (ohtn.on.ca)](https://www.ohtn.on.ca/rapid-response-the-impact-of-location-on-implementation-of-hivsti-prevention-interventions-among-lgbtq-communities/)

[A cross-jurisdictional review of HIV testing intervals for population groups at high risk of HIV infection – The Ontario HIV Treatment Network (ohtn.on.ca)](https://www.ohtn.on.ca/rapid-response-a-cross-jurisdictional-review-of-hiv-testing-intervals-for-population-groups-at-high-risk-of-hiv-infection/)

[Linking sexual compulsivity and HIV transmission – The Ontario HIV Treatment Network (ohtn.on.ca)](https://www.ohtn.on.ca/rapid-response-5-linking-sexual-compulsivity-and-hiv-transmission/)

[RR175-Doxycycline-prophylaxis-for-bacterial-STIs.pdf (ohtn.on.ca)](https://www.ohtn.on.ca/wp-content/uploads/2023/03/RR175-Doxycycline-prophylaxis-for-bacterial-STIs.pdf)

[Testing interventions for HIV and sexually transmitted infections (STIs) among young men who have sex with men – The Ontario HIV Treatment Network (ohtn.on.ca)](https://www.ohtn.on.ca/rapid-response-testing-interventions-for-hiv-and-sexually-transmitted-infections-among-young-men-who-have-sex-with-men/)

**Catie**

site:www.catie.ca AND (transgender OR trans) AND ("sexually transmitted" OR STI OR STD OR HIV OR herpes OR hepatitis OR syphilis OR gonorrhea OR gonorrhoea OR chlamydia OR scabies OR vaginitis OR urethritis)

About 1,950 results

Done using InPrivate window in Microsoft Edge

Date: October 5, 2023

[What is the prevalence of HIV in trans people? | CATIE - Canada's source for HIV and hepatitis C information](https://www.catie.ca/prevention-in-focus/what-is-the-prevalence-of-hiv-in-trans-people)

[HIV prevention and trans people: What the Trans PULSE Project can tell us | CATIE - Canada's source for HIV and hepatitis C information](https://www.catie.ca/prevention-in-focus/hiv-prevention-and-trans-people-what-the-trans-pulse-project-can-tell-us)

[What are the barriers and facilitators to HIV PrEP use among transgender people? | CATIE - Canada's source for HIV and hepatitis C information](https://www.catie.ca/prevention-in-focus/what-are-the-barriers-and-facilitators-to-hiv-prep-use-among-transgender-people)

[Pre-exposure prophylaxis (PrEP) | CATIE - Canada's source for HIV and hepatitis C information](https://www.catie.ca/pre-exposure-prophylaxis-prep-0)

**Action Canada for Sexual Health & Rights**

site:www.actioncanadashr.org AND (transgender OR trans) AND ("sexually transmitted" OR STI OR STD OR HIV OR herpes OR hepatitis OR syphilis OR gonorrhea OR gonorrhoea OR chlamydia OR scabies OR vaginitis OR urethritis)

About 142 results

Done using InPrivate window in Microsoft Edge

Date: October 6, 2023

[WHO Policy brief: Transgender people and HIV | Action Canada for Sexual Health and Rights (actioncanadashr.org)](https://www.actioncanadashr.org/resources/policy-briefs-submissions/2019-04-10-who-policy-brief-transgender-people-and-hiv)

[What does “protecting children” actually look like? | Action Canada for Sexual Health and Rights (actioncanadashr.org)](https://www.actioncanadashr.org/resources/sexual-health-hub/sex-ed/what-does-protecting-children-actually-look)

[HESA report.pdf (actioncanadashr.org)](https://www.actioncanadashr.org/sites/default/files/2019-04/HESA%20report.pdf)

[Action Canada for Sexual Health & Rights (actioncanadashr.org)](https://www.actioncanadashr.org/sites/default/files/2019-03/Ontario-CSE-submission-FINAL.pdf)

**Community-Based Research Centre**

site:www.cbrc.net AND (transgender OR trans) AND ("sexually transmitted" OR STI OR STD OR HIV OR herpes OR hepatitis OR syphilis OR gonorrhea OR gonorrhoea OR chlamydia OR scabies OR vaginitis OR urethritis)

About 706 results

Done using InPrivate window in Microsoft Edge

Date: October 6, 2023

[Federal LGBT2Q Health Study - Community-Based Research Centre (cbrc.net)](https://www.cbrc.net/hesa)

[Highlights - Engage (engage-men.ca)](https://www.engage-men.ca/our-work/highlights/)

[Identifying Health Differences Between Transgender and Cisgender Gay, Bisexual and Other Men Who Have Sex With Men Using a Community-Based Approach - Community-Based Research Centre (cbrc.net)](https://www.cbrc.net/identifying_health_differences_between_transgender_and_cisgender_gay_bisexual_and_other_men_who_have_sex_with_men_using_a_community_based_approach)

[Barriers to PrEP Uptake in Two Spirit, Gay, Bisexual, Trans and Queer Men, and Non-Binary People in Canada - Community-Based Research Centre (cbrc.net)](https://www.cbrc.net/barriers_to_prep_uptake)

[Short Oral Presentations — Trans Health - Community-Based Research Centre (cbrc.net)](https://www.cbrc.net/short_oral_presentations_trans_health)

[Affirming Trans Health and Wellness - Community-Based Research Centre (cbrc.net)](https://www.cbrc.net/affirming_trans_health_and_wellness)

**Gay Men’s Sexual Health Alliance (GMSH)**

site:https://gmsh.ca AND (transgender OR trans) AND ("sexually transmitted" OR STI OR STD OR HIV OR herpes OR hepatitis OR syphilis OR gonorrhea OR gonorrhoea OR chlamydia OR scabies OR vaginitis OR urethritis)

About 99 results

Done using InPrivate window in Microsoft Edge

Date: October 10, 2023

[GMSH_TechnicalReportDraft_2021_V01_03.pdf](https://gmsh.ca/wp-content/uploads/2022/04/GMSH_TechnicalReportDraft_2021_V01_03.pdf)

**BC Hepatitis Network Society**

site:http://bchep.org AND (transgender OR trans) AND ("sexually transmitted" OR STI OR STD OR HIV OR herpes OR hepatitis OR syphilis OR gonorrhea OR gonorrhoea OR chlamydia OR scabies OR vaginitis OR urethritis)

About 3 results

Done using InPrivate window in Microsoft Edge

Date: October 10, 2023

No relevant results

**Canadian Aboriginal AIDS Network (CAAN)**

site:https://caan.ca AND (transgender OR trans) AND ("sexually transmitted" OR STI OR STD OR HIV OR herpes OR hepatitis OR syphilis OR gonorrhea OR gonorrhoea OR chlamydia OR scabies OR vaginitis OR urethritis)

About 76 results

Done using InPrivate window in Microsoft Edge

Date: October 10, 2023

[Microsoft Word - reprint_cover_page.doc (caan.ca)](https://caan.ca/wp-content/uploads/2021/05/Vol-2.-Article-4..pdf)

[Hogg - research and findings (caan.ca)](https://caan.ca/wp-content/uploads/2021/08/Working-Together-Hogg.pdf)

[Vol-4.-Article-1..pdf (caan.ca)](https://caan.ca/wp-content/uploads/2021/05/Vol-4.-Article-1..pdf)

[Resilience among two-spirit males who have been living with HIV long term Findings from a scoping review (caan.ca)](https://caan.ca/wp-content/uploads/2021/11/Resilience-among-two-spirit-males-who-have-been-living-with-HIV-long-term-Findings-from-a-scoping-review.pdf)

[April 2019 Newsblast FINAL (caan.ca)](https://caan.ca/wp-content/uploads/2021/05/April-2019.pdf)

[Hogg FR (caan.ca)](https://caan.ca/wp-content/uploads/2021/10/Travailler-ensemble-allie%CC%81s-dans-la-recherche.pdf)

[Pruden FR (caan.ca)](https://caan.ca/wp-content/uploads/2021/10/Facteurs-associe%CC%81s-aux-connaissances-en-matie%CC%80re-de-sante%CC%81-sexuelle.pdf)

[Vol 5 Beswick FR (caan.ca)](https://caan.ca/wp-content/uploads/2021/05/Vol-5.-Article-5-FR.pdf)

[Resilience-chez-les-hommes-bispirituels-qui-vivent-avec-le-VIH-depuis-longtemps-.pdf (caan.ca)](https://caan.ca/wp-content/uploads/2021/12/Resilience-chez-les-hommes-bispirituels-qui-vivent-avec-le-VIH-depuis-longtemps-.pdf)

**Second set of search terms**

| Date | Organization Name | Specific pages flagged as relevant for checking |
| --- | --- | --- |
| Oct 5 | REACH Nexus | No relevant results |
| Oct 5 | Ontario HIV Treatment Network | No relevant results |
| Oct 5 | Catie | 5 pages flagged – See below. |
| Oct 5 | Action Canada SHR | No relevant results |
| Oct 6 | CBRC | No relevant results |
| Oct 10 | Gay Mens Sexual Health Alliance | No relevant results |
| Oct 10 | BC Hepatitis C Network | No relevant results |
| Oct 10 | Canadian Aboriginal AIDS Network (CAAN) | No relevant results |

**SET #2**

**REACH Nexus**

site:www.reachnexus.ca AND (transgender OR trans) AND (“human papillomavirus virus” OR trichomonas OR “granuloma inguinale” OR “bacterial vaginosis” OR “pelvic inflammatory disease” OR proctitis OR phthirus OR “human T-lymphotropic virus” OR epididymitis OR “molluscum contagiosum”)

0 RESULTS

Done using InPrivate window in Microsoft Edge

Date: October 5, 2023

**Ontario HIV Treatment Network**

site:www.ohtn.on.ca AND (transgender OR trans) AND (“human papillomavirus virus” OR trichomonas OR “granuloma inguinale” OR “bacterial vaginosis” OR “pelvic inflammatory disease” OR proctitis OR phthirus OR “human T-lymphotropic virus” OR epididymitis OR “molluscum contagiosum”)

10 results

Done using InPrivate window in Microsoft Edge

Date: October 5, 2023

No new relevant results

**Catie**

site:www.catie.ca AND (transgender OR trans) AND (“human papillomavirus virus” OR trichomonas OR “granuloma inguinale” OR “bacterial vaginosis” OR “pelvic inflammatory disease” OR proctitis OR phthirus OR “human T-lymphotropic virus” OR epididymitis OR “molluscum contagiosum”)

About 26 results

Done using InPrivate window in Microsoft Edge

Date: October 5, 2023

[64-02-14-1200-STI-Report-2011_EN-FINAL.pdf (catie.ca)](https://www.catie.ca/sites/default/files/64-02-14-1200-STI-Report-2011_EN-FINAL.pdf)

[Report-on-STIs-in-Canada-2012.pdf (catie.ca)](https://www.catie.ca/sites/default/files/Report-on-STIs-in-Canada-2012.pdf)

[2009 Report on STI in Canada_EN.pdf (catie.ca)](https://www.catie.ca/sites/default/files/2009%20Report%20on%20STI%20in%20Canada_EN.pdf)

[Chlamydia-among-young-women.pdf (catie.ca)](https://www.catie.ca/sites/default/files/Chlamydia-among-young-women.pdf)

[3382_CATIE_CarolStrike_BestPracticeRecommendations_2021-EN-Final.pdf](https://www.catie.ca/sites/default/files/2021-11/3382_CATIE_CarolStrike_BestPracticeRecommendations_2021-EN-Final.pdf)

**Action Canada for Sexual Health & Rights**

site:www.actioncanadashr.org AND (transgender OR trans) AND (“human papillomavirus virus” OR trichomonas OR “granuloma inguinale” OR “bacterial vaginosis” OR “pelvic inflammatory disease” OR proctitis OR phthirus OR “human T-lymphotropic virus” OR epididymitis OR “molluscum contagiosum”)

About 2 results

Done using InPrivate window in Microsoft Edge

Date: October 5, 2023

No relevant results

**Community-Based Research Centre**

site:www.cbrc.net AND (transgender OR trans) AND (“human papillomavirus virus” OR trichomonas OR “granuloma inguinale” OR “bacterial vaginosis” OR “pelvic inflammatory disease” OR proctitis OR phthirus OR “human T-lymphotropic virus” OR epididymitis OR “molluscum contagiosum”)

0 RESULTS WITH THIS SEARCH

Done using InPrivate window in Microsoft Edge

Date: October 6, 2023

**Gay Men’s Sexual Health Alliance (GMSH)**

site:https://gmsh.ca AND (transgender OR trans) AND (“human papillomavirus virus” OR trichomonas OR “granuloma inguinale” OR “bacterial vaginosis” OR “pelvic inflammatory disease” OR proctitis OR phthirus OR “human T-lymphotropic virus” OR epididymitis OR “molluscum contagiosum”)

About 1 results

Done using InPrivate window in Microsoft Edge

Date: October 10, 2023

No relevant results

**BC Hepatitis Network Society**

site:http://bchep.org AND (transgender OR trans) AND (“human papillomavirus virus” OR trichomonas OR “granuloma inguinale” OR “bacterial vaginosis” OR “pelvic inflammatory disease” OR proctitis OR phthirus OR “human T-lymphotropic virus” OR epididymitis OR “molluscum contagiosum”)

0 RESULTS

Done using InPrivate window in Microsoft Edge

Date: October 10, 2023

**Canadian Aboriginal AIDS Network (CAAN)**

site:https://caan.ca AND (transgender OR trans) AND (“human papillomavirus virus” OR trichomonas OR “granuloma inguinale” OR “bacterial vaginosis” OR “pelvic inflammatory disease” OR proctitis OR phthirus OR “human T-lymphotropic virus” OR epididymitis OR “molluscum contagiosum”)

0 RESULTS

Done using InPrivate window in Microsoft Edge

Date: October 10, 2023

**Third set of search terms**

| Date | Organization Name | Specific pages flagged as relevant for checking |
| --- | --- | --- |
| Oct 5 | REACH Nexus | No relevant results |
| Oct 5 | Ontario HIV Treatment Network | 3 pages flagged – See below. |
| Oct 5 | Catie | 5 pages flagged – See below. |
| Oct 5 | Action Canada SHR | 1 page flagged – See below. |
| Oct 6 | CBRC | [1](https://www.cbrc.net/challenges_opportunities_and_priorities) page flagged – See below. |
| Oct 10 | Gay Mens Sexual Health Alliance | No relevant results |
| Oct 10 | BC Hepatitis C Network | No relevant results |
| Oct 10 | Canadian Aboriginal AIDS Network (CAAN) | No relevant results |

**SET #3**

**REACH Nexus**

site:www.reachnexus.ca AND (transgender OR trans) AND ("STBBI" OR "STBBIs")

About 7 results

Done using InPrivate window in Microsoft Edge

Date: October 5, 2023

No relevant results

**Ontario HIV Treatment Network**

site:www.ohtn.on.ca AND (transgender OR trans) AND ("STBBI" OR "STBBIs")

About 23 results

Done using InPrivate window in Microsoft Edge

Date: October 5, 2023

[View from the Front Lines, 2017 (ohtn.on.ca)](https://www.ohtn.on.ca/wp-content/uploads/view-from-the-frontlines/VFFL-2017-EN.pdf)

[vffl2019-english-book.pdf (ohtn.on.ca)](https://www.ohtn.on.ca/wp-content/uploads/2019/07/vffl2019-english-book.pdf)

[The Current State of the HIV Epidemic among Aboriginal People in Ontario (ohtn.on.ca)](https://www.ohtn.on.ca/wp-content/uploads/2014/09/Indigenous-Report-2014Final.pdf)

**Catie**

site:www.catie.ca AND (transgender OR trans) AND ("STBBI" OR "STBBIs")

About 65 results

Done using InPrivate window in Microsoft Edge

Date: October 5, 2023

[Challenges, opportunities and priorities: Findings from a national consultation on HIV and STBBI prevention among gay, bisexual, trans, two-spirited and queer men (GBT2Q) in Canada | CATIE - Canada's source for HIV and hepatitis C information](https://www.catie.ca/resource/challenges-opportunities-and-priorities-findings-from-a-national-consultation-on-hiv-and)

[QA-STI-EN-FINAL.pdf (catie.ca)](https://www.catie.ca/sites/default/files/QA-STI-EN-FINAL.pdf)

[Syphilis-among-gay-bisexual-two-spirit-and-other-MSM.pdf (catie.ca)](https://www.catie.ca/sites/default/files/Syphilis-among-gay-bisexual-two-spirit-and-other-MSM.pdf)

[Population-Specific HIV/AIDS Status Report - Aboriginal People (catie.ca)](https://www.catie.ca/sites/default/files/26344.pdf)

[SR-Gay-Bisexual-Two-Spirit-and-other-Men-Who-Have-Sex-With-Men.pdf (catie.ca)](https://www.catie.ca/sites/default/files/SR-Gay-Bisexual-Two-Spirit-and-other-Men-Who-Have-Sex-With-Men.pdf)

**Action Canada for Sexual Health & Rights**

site:www.actioncanadashr.org AND (transgender OR trans) AND ("STBBI" OR "STBBIs")

About 20 results

Done using InPrivate window in Microsoft Edge

Date: October 6, 2023

[Action Canada Election 2021 Brief.pdf (actioncanadashr.org)](https://www.actioncanadashr.org/sites/default/files/2021-09/Action%20Canada%20Election%202021%20Brief.pdf)

**Community-Based Research Centre**

site:www.cbrc.net AND (transgender OR trans) AND ("STBBI" OR "STBBIs")

About 119 results

Done using InPrivate window in Microsoft Edge

Date: October 6, 2023

[Challenges, Opportunities and Priorities (cbrc.net)](https://www.cbrc.net/challenges_opportunities_and_priorities)

**Gay Men’s Sexual Health Alliance (GMSH)**

site:https://gmsh.ca AND (transgender OR trans) AND ("STBBI" OR "STBBIs")

About 2 results

Done using InPrivate window in Microsoft Edge

Date: October 10, 2023

No relevant results.

**BC Hepatitis Network Society**

site:http://bchep.org AND (transgender OR trans) AND ("STBBI" OR "STBBIs")

0 RESULTS

Done using InPrivate window in Microsoft Edge

Date: October 10, 2023

**Canadian Aboriginal AIDS Network (CAAN)**

site:https://caan.ca AND (transgender OR trans) AND ("STBBI" OR "STBBIs")

About 38 results

Done using InPrivate window in Microsoft Edge

Date: October 10, 2023

No new relevant results
