# Supplementary material for: Sexually transmitted and blood-borne infections in transgender and non-binary people in Canada: A scoping review
Source: PLoS One. 2025 May 20;20(5):e0322521. doi: 10.1371/journal.pone.0322521 (PMC12091819; doi:10.1371/journal.pone.0322521)
Supplement: S2 Table — (DOCX) [file pone.0322521.s002.docx]

**S2 Table: Reasons for exclusion for studies identified via databases**

| **Authors** | **Year** | **Title** | **Journal** | **Reason for Exclusion** |
| --- | --- | --- | --- | --- |
| Armstrong H | 2017 | The momentum health study: Understanding the context of HIV treatment as prevention among gay, bisexual, and other men who have sex with men in Vancouver, Canada | The Journal of Sexual Medicine | Abstract, author had no more details |
| Bauer & Hammond | 2015 | Toward a broader conceptualization of trans women's sexual health | The Canadian Journal of Human Sexuality | Ineligible outcome |
| Bauer et al. | 2013 | Sexual Health of Trans Men Who Are Gay, Bisexual, or Who Have Sex with Men: Results from Ontario, Canada | International Journal of Transgenderism | Duplicate |
| Bauer et al. | 2015 | Corrigendum: [ Sexual Health of Trans Men Who Are Gay, Bisexual, or Who Have Sex with Men: Results from Ontario, Canada] | International Journal of Transgenderism | Ineligible outcome |
| Davy-Mendez et al | 2021 | Racial, ethnic, and gender disparities in hospitalizations among persons with HIV in the United States and Canada, 2005-2015 | AIDS | No disaggregated Canada data |
| Deering et al | 2018 | Social-structural correlates of HIV stigma among women living with HIV in Metro Vancouver | Journal of the International AIDS Society | Duplicate |
| Deering et al | 2021 | Social and structural barriers to primary care access among women living with HIV in metro Vancouver, Canada: A longitudinal cohort study | The Journal of the Association of Nurses in AIDS Care | Ineligible outcome |
| Deering et al | 2021 | Prevalence and correlates of HIV stigma among women living with HIV in metro Vancouver, Canada | AIDS and Behavior | Ineligible outcome |
| Gahagan et al | 2014 | The national consensus statement on women, trans people and girls and HIV research in Canada : Recommendations for social science researchers | Canadian Journal of Infectious Diseases and Medical Microbiology | Abstract, received more details |
| Hoornenborg et al | 2017 | Pre-exposure prophylaxis for MSM and transgender persons in early adopting countries | AIDS | No disaggregated Canada data |
| Howard PA | 2014 | The depth of water requires knowledge: Listening to the voices of the HIV patient journey | Canadian Journal of Infectious Diseases and Medical Microbiology | Abstract, received more details |
| Kaida et al | 2018 | High mortality among women living with HIV enrolled in Canada's largest community-based cohort study | Journal of the International AIDS Society | Abstract, received more details |
| Kruse et al | 2022 | Care of sexual and gender minorities in the emergency department: A scoping review | Annals of Emergency Medicine | Literature Review |
| Lachowsky et al | 2020 | Prevalence, trends and correlates of HIV pre-exposure prophylaxis (PrEP) use during sexual events by sexual minority men in Canada's three largest metropolitan areas | Journal of the International AIDS Society | Abstract, author had no more details |
| Lacombe-Duncan et al | 2019 | Gender-affirming healthcare experiences and medical transition among transgender women living with HIV: A mixed-methods study | Sexual Health | Ineligible outcome |
| Lacombe-Duncan et al | 2021 | Mental health among transgender women living with HIV in Canada: findings from a national community-based research study | AIDS Care - Psychological and Socio-Medical Aspects of AIDS/HIV | Duplicate |
| Lacombe-Duncan A | 2019 | Understanding access to HIV-related and gender-affirming healthcare for trans women with HIV in Canada: A mixed methods study | Dissertation Abstracts International Section A: Humanities and Social Sciences (Published by UofT in 2018) | Duplicate |
| Lacombe-Duncan & Olawale | 2022 | Context, types, and consequences of violence across the life course: a qualitative study of the lived experiences of transgender women living with HIV | Journal of Interpersonal Violence | Ineligible outcome |
| Liboro et al | 2022 | Kinky sex and deliberate partner negotiations: Case studies of Canadian transgender men who have sex with men, their HIV risks, safer sex practices, and prevention needs | International Journal of Environmental Research and Public Health | Case study |
| Logie et al | 2019 | “Automatic assumption of your gender, sexuality and sexual practices is also discrimination”: Exploring sexual healthcare experiences and recommendations among sexually and gender diverse persons in Arctic Canada | Health and Social Care in the Community | Ineligible outcome |
| Logie et al | 2018 | ‘In the North you can’t be openly gay’: Contextualising sexual practices among sexually and gender diverse persons in Northern Canada | Global Public Health | Ineligible outcome |
| Lyons et al | 2015 | "it depends on how you represent yourself in public": Transgender individuals living with and affected by HIV and their encounters with frontline HIV services | Canadian Journal of Infectious Diseases and Medical Microbiology | Abstract, received more details |
| Lyons et al | 2013 | Transgender, transsexual and two-spirit individuals who use drugs: Stigma, violence, and HIV vulnerabilities | Canadian Journal of Infectious Diseases and Medical Microbiology | Abstract, author had no more details |
| Namaste VK | 2014 | HIV/AIDS and female-to-male transsexuals and transvestites: Results from a needs assessment in Quebec | Chapter in *Transgender and HIV.* Book was first published in 2001. | Published before 2013 |
| Parsons JT | 2014 | Contemporary research on sex work | Book. First published in 2005. | Published before 2013 |
| Pearson et al | 2022 | Sex work community participation in criminalized environments: A community-based cohort study of occupational health impacts in Vancouver, Canada: 2010–2019 | International Journal for Equity in Health | Ineligible outcome |
| Pico Espinosa et al | 2021 | PrEP-related stigma and PrEP status among gay, bisexual and other men who have sex with men, and transgender women in Ontario and British Columbia, Canada | HIV Medicine | Abstract, received more details |
| Poteat et al | 2020 | Characterizing the human immunodeficiency virus care continuum among transgender women and cisgender women and men in clinical care: A retrospective time-series analysis | Clinical Infectious Diseases | No disaggregated Canada data |
| Rana et al | 2022 | Community perspectives on ideal bacterial STI testing services for gay, bisexual, and other men who have sex with men in Toronto, Canada: a qualitative study | BMC Health Services Research | Case study/short case series |
| Ranjan et al | 2018 | Barriers and facilitators to hepatitis B immunization among sex workers: Implications for integrated HIV, STI, and viral hepatitis services | Journal of viral hepatitis | Abstract, received more details |
| Reisner & Murchison | 2016 | A global research synthesis of HIV and STI biobehavioural risks in female-to-male transgender adults | Global Public Health | Literature review |
| Rich et al | 2021 | Development of a computable phenotype to identify a transgender sample for health research purposes: A feasibility study in a large linked provincial healthcare administrative cohort in British Columbia, Canada | BMJ Open | Ineligible outcome |
| Rich et al | 2015 | Making sense of the inclusion of transgender men and their HIV risk profile within a biobehavioural population study of gay and other men who have sex with men (MSM) in Vancouver, British Columbia | Canadian Journal of Infectious Diseases and Medical Microbiology | Abstract, received more details |
| Rios V | 2022 | Transgender men who have sex with men: Sexual risk behaviors and multiple minority stressors | Dissertation Abstracts International: Section B: The Sciences and Engineering (Published by Palo Alto University in 2020/2021) | No disaggregated Canada data |
| Scheim et al | 2014 | "I didn't get the feeling that they knew what they were doing": HIV/STI testing experiences of trans men who have sex with men in Ontario | Canadian Journal of Infectious Diseases and Medical Microbiology | Duplicate |
| Scheim et al | 2015 | Factors associated with HIV risk in Ontario's broad transfeminine population: The challenge of heterogeneity for transgender HIV prevention research | Canadian Journal of Infectious Diseases and Medical Microbiology | Abstract, received more details |
| Scruton et al | 2014 | A national needs assessment of trans people | Canadian Journal of Infectious Diseases and Medical Microbiology | Abstract, received more details |
| Sernick et al | 2022 | In the midst of plenty: Experiences of food insecurity amongst women living with HIV in Vancouver, Canada | Health and Social Care in the Community | No disaggregated transgender data |
| Socías et al | 2015 | Gaps in the hepatitis C continuum of care among sex workers in Vancouver, British Columbia: Implications for voluntary hepatitis C virus testing, treatment and care | Canadian Journal of Gastroenterology and Hepatology | No disaggregated transgender data |
| Sullivan et al | 2014 | The global north: HIV epidemiology in high-income countries | Current Opinion in HIV and AIDS | No disaggregated transgender data |
| Thornhill et al | 2022 | Human monkeypox virus infection in women and non-binary individuals during the 2022 outbreaks: a global case series | The Lancet | No disaggregated Canada data |
| Veale et al | 2016 | Prevalence of pregnancy involvement among Canadian transgender youth and its relation to mental health, sexual health, and gender identity | International Journal of Transgenderism | Ineligible outcome |
| Ware et al | 2014 | When the rainbow ain't enough: Intersectionality and HIV prevention for diverse trans MSM communities | Canadian Journal of Infectious Diseases and Medical Microbiology | Abstract, couldn’t contact author |
| Winkelman et al | 2022 | Pharmacist-delivered HIV point-of-care testing in Ontario: Lessons learned from the GetaTest pilot | Canadian Pharmacists Journal | No disaggregated transgender data |
| Zeggagh et al | 2022 | Incidence and risk factors for recurrent sexually transmitted infections among MSM on HIV pre-exposure prophylaxis | AIDS | No disaggregated transgender data |
| Zhang et al | 2021 | Prevalence and correlates of having sexual and reproductive health priorities met by HIV providers among women living with HIV in a Canadian setting | Sexual and Reproductive Healthcare | Ineligible outcome |

| Ineligible outcome | n = 12 |  |  |
| --- | --- | --- | --- |
| Abstract, received more details | n = 9 |  |  |
| No disaggregated transgender data | n = 5 |  |  |
| Duplicate | n = 5 |  |  |
| No disaggregated Canada data | n = 5 |  |  |
| Abstract, author had no more details | n = 3 |  |  |
| Published before 2013 | n = 2 |  |  |
| Case study | n = 2 |  |  |
| Literature review | n = 2 |  |  |
| Abstract, couldn’t contact author | n = 1 |  |  |
| **Total** | **n = 46** |  |  |
