# Supplementary material for: Sexually transmitted and blood-borne infections in transgender and non-binary people in Canada: A scoping review
Source: PLoS One. 2025 May 20;20(5):e0322521. doi: 10.1371/journal.pone.0322521 (PMC12091819; doi:10.1371/journal.pone.0322521)
Supplement: Attachment — Submitted filename: Response to Reviewers.docx [file pone.0322521.s005.docx]

**We thank the reviewer for their helpful comments.**

Review Comments to the Author

Reviewer #1: Strength of Manuscript: The article addresses a relevant and topical issue on STBBIs (sexually transmitted infections and other reproductive tract infections) in transgender and non-binary people in Canada. The study incorporates a scoping analysis, which was complemented by interviews with 18 experts, including researchers and people working in transgender community organizations. The methodological approach is comprehensive and appropriate for the topic.

Relevance of the Data and Conclusions: The data collected is consistent and supports the conclusions presented. The authors highlight the need for more robust and inclusive data to improve resources, treatment and prevention efforts for these populations, which is in line with the guidelines of the 2SLGBQTI+ Federal Action Plan. In addition, the analysis identifies significant gaps in the current literature, most notably the lack of disaggregated data that considers intersectional aspects such as race, ethnicity, disability and age, which can affect the risk of STBBI.

Recommendations for Improvement: The article could be strengthened with a more detailed analysis of sexual behaviors and their impacts on the health of transgender and non-binary subpopulations.

**We agree that such an analysis would be valuable but due to limitations in the data we found, we cannot comment on how sexual behaviours impact STBBI outcomes. In the discussion, we have added the second sentence below to make this clear: “The findings of this review suggest that different sub-populations of transgender and non-binary people have different levels of risk of contracting STBBIs, in part because they have different levels of sexual activity, and access testing, treatment and prevention services at different rates. However, none of the studies we found collected STBBI prevalence data alongside data relating to behavioural risk exposures and engagement in prevention and care, making it difficult to determine precisely how sexual risk exposures impact STBBI risk in these populations.”**

In addition, the inclusion of data on the effects of gender affirmation surgery and other intersections between social identity and health would help to further refine the conclusions.

**Unfortunately, we did not find any data on the effects of gender-affirming surgery on STBBI risk. We have added the following sentence to the discussion to make this clear: “We found no data on how gender-affirming surgery affects STBBI risk.” Also, we are unable to comment on the effects of other intersections between social identity and health because, as we had written, “the studies we included in this review did not provide data disaggregated on intersecting aspects of identity such as race and ethnicity, disability and age that may affect STBBI risk.”**

It would also be beneficial if the authors included specific recommendations for public policy and health practices based on the study's findings.

**We thank the reviewer for the helpful prompt to consider how the evidence synthesised in this scoping review can inform public health. The available evidence is insufficient to make specific recommendations, but this is an important conclusion of our review. We have added the following sentence to the summary paragraph: “It is not possible to make specific recommendations for public policy based on the evidence as it currently exists.” We have added the same sentence to the abstract to highlight its importance and consequently edited the abstract to stay within the word count.**

Ethical Considerations and Conflicts of Interest: The article declares no conflicts of interest among the authors, which suggests an unbiased analysis. There is no indication that the study involved the need for ethical approval, presumably because the data was collected secondarily or through non-clinical interviews.

**This is correct, we did not require ethical approval for this work.**

In summary, the manuscript is technically sound and offers significant contributions to the field. With minor revisions and expansions, it could become an essential piece to guide future health policies and practices for transgender and non-binary people in Canada.

Journal Requirements:

When submitting your revision, we need you to address these additional requirements.

1. Please ensure that your manuscript meets PLOS ONE's style requirements, including those for file naming. The PLOS ONE style templates can be found at 
<https://journals.plos.org/plosone/s/file?id=wjVg/PLOSOne_formatting_sample_main_body.pdf> and 
<https://journals.plos.org/plosone/s/file?id=ba62/PLOSOne_formatting_sample_title_authors_affiliations.pdf>

**We’ve made changes based on these requirements.**

2. We note that your Data Availability Statement is currently as follows: All relevant data are within the manuscript and its Supporting Information files.

Please confirm at this time whether or not your submission contains all raw data required to replicate the results of your study. Authors must share the “minimal data set” for their submission. PLOS defines the minimal data set to consist of the data required to replicate all study findings reported in the article, as well as related metadata and methods (https://journals.plos.org/plosone/s/data-availability#loc-minimal-data-set-definition).

For example, authors should submit the following data:

- The values behind the means, standard deviations and other measures reported;
- The values used to build graphs;
- The points extracted from images for analysis.

Authors do not need to submit their entire data set if only a portion of the data was used in the reported study.

If your submission does not contain these data, please either upload them as Supporting Information files or deposit them to a stable, public repository and provide us with the relevant URLs, DOIs, or accession numbers. For a list of recommended repositories, please see <https://journals.plos.org/plosone/s/recommended-repositories>.

If there are ethical or legal restrictions on sharing a de-identified data set, please explain them in detail (e.g., data contain potentially sensitive information, data are owned by a third-party organization, etc.) and who has imposed them (e.g., an ethics committee). Please also provide contact information for a data access committee, ethics committee, or other institutional body to which data requests may be sent. If data are owned by a third party, please indicate how others may request data access.

**Yes, the submission contains all raw data required to replicate the results of our study. The submission is a scoping review. All included papers or grey literature are publicly available, including all the data we extracted as part of our review, and we have included the full search terms we used in the Supporting Material.**

3. Please include captions for your Supporting Information files at the end of your manuscript, and update any in-text citations to match accordingly. Please see our Supporting Information guidelines for more information: <http://journals.plos.org/plosone/s/supporting-information>.

**This has been done.**

4. We note that this manuscript is a systematic review or meta-analysis; our author guidelines therefore require that you use PRISMA guidance to help improve reporting quality of this type of study. Please upload copies of the completed PRISMA checklist as Supporting Information with a file name “PRISMA checklist”.

**This has been done.**

5. Please review your reference list to ensure that it is complete and correct. If you have cited papers that have been retracted, please include the rationale for doing so in the manuscript text, or remove these references and replace them with relevant current references. Any changes to the reference list should be mentioned in the rebuttal letter that accompanies your revised manuscript. If you need to cite a retracted article, indicate the article’s retracted status in the References list and also include a citation and full reference for the retraction notice.

**We have checked the reference list, which is complete and correct, and we also checked and confirmed that none of the papers have been retracted.**
