# Supplementary material for: Sexually transmitted and blood-borne infections in transgender and non-binary people in Canada: A scoping review
Source: PLoS One. 2025 May 20;20(5):e0322521. doi: 10.1371/journal.pone.0322521 (PMC12091819; doi:10.1371/journal.pone.0322521)
Supplement: Attachment — Submitted filename: Response_to_Reviewers_auresp_2.docx [file pone.0322521.s006.docx]

Many thanks for this revised manuscript which is of high quality and meets publication criteria.

I would recommend a further minor revision in line with Reviewer 2's comments to include the interviews of 18 experts in the methodology and results, and if applicable, expanded in the discussion. This would allow the reader to understand how these fit with the data and if further work from this is planned.

==============================

Reviewer #2: This article is incredibly well thought out and strategically planned in order to capture and meet its aims of understanding the available data relating to STBBIs in transgender and non-binary people in Canada. It not only clearly identifies and analyses the data that is available but also importantly identifies gaps in research and publication that can be used to guide future research and data collection that could be used to inform care and sexual health services in a deliberate and meaningful way to serve people with gender expansive identities. Notably there is a clear identification of a scarcity of disaggregated data and therefore, the limitations in application of some the current data. The analysis of the differences in patterns of behaviours of people with different gender identities and sexual orientations along where there are gaps in this data was done with inclusion of the nuances and specifics that can shape this.

The article is insightful and relevant. The scoping analysis is comprehensive and appropriate and demonstrates methodology that is well fitting in exploring this topic where there are such inconsistencies and variations in how data is collected and defined. I commend the authors highly and acknowledge the dedication and quality of this work.
The data collected is put into perspective and linked clearly with the conclusions and discussions and where this fits with future work.

In the discussion section there was a very useful section regarding the interviews with 18 experts which brought important framing to the current climate for transgender and non-binary people in the context of sexual health, however, I wonder why these interviews were not mentioned throughout the article, specifically in the methodology or results and only feature in the discussion as a few sentences. They could be expanded and put into conversation with the data collected in this article or it be indicated how the data from these interviews could be used for additional work on this topic.

Irrespective of this, I think this is a formidable piece of work that could be pivotal in guiding future work for the promotion of improved sexual health outcomes for gender expansive individuals navigating differing intersections of unmet need.

**Thank you for your kind comments.**

**We have followed your advice and included more details of the interviews in both the methods and results sections.**

**In the methods section, we have written:**

**“Alongside our scoping review, we also interviewed a convenience sample of 18 experts on how STBBIs affect transgender and non-binary people in Canada. The aim of the interviews was to understand their views on the state of the field and to learn about recent and ongoing research on the topic. Twelve (67%) of the experts were academic researchers, five (28%) worked with transgender-focused community organisations and one (6%) worked in government. Around half of the experts identified as transgender or non-binary.**

**Interviews were semi-structured. We asked the experts for their views on the state of STBBI research and surveillance among transgender and non-binary people in Canada; about data gaps in the field; about recent and ongoing research in the field; and for suggestions for future work they thought would be valuable. We prepared an interview guide that asked broadly about these topics but discussions were largely guided by the responses of the experts towards the topics they felt were most important in the field. We chose this interview strategy because it mirrored the search strategy of our scoping review, which was deliberately broad and exploratory, aiming to identify data on a range of factors affecting STBBI epidemiology and care in these populations without pre-specifying precise outcomes we were searching for. Interviews were conducted by two authors (JB and MB), in some cases in conjunction with a third author (JA), between December 2023 and April 2024. Interviews lasted between 30 and 60 minutes and were conducted virtually.”**

**In the results section, we have written:**

***“Findings from expert interviews***

**All experts agreed that more data are needed on STBBIs in transgender and non-binary people in Canada. A common theme discussed across interviews was the lack of STBBI surveillance data disaggregated by gender identity at the national and provincial and territorial levels. As a result, most prevalence data and other epidemiological information come from research studies focusing on transgender people, studies which were not designed to produce generalisable prevalence estimates and which are highly limited both geographically and in terms of sample size. In several interviews, experts also noted a data gap relating to how STBBI risk is affected by sexual anatomy and gender-affirming surgery and by the intersection of physiology, identity, social determinants of health and sexual behaviour.**

**One expert suggested that sexual inactivity is relatively common in transgender communities in Canada and that future surveys in these populations should have behavioural entry criteria such as having had sex in the past six months. Some experts thought that existing transgender-specific studies conducted in Canada, such as Trans PULSE Ontario, did not sufficiently capture the subpopulations most at risk of STBBIs, and advocated for future studies focusing on these subpopulations. However, opinions varied on the subpopulations most at risk. Some saw transgender women, transgender GBMSM and transgender and non-binary people who are part of gay sex networks as being at particular risk. Others thought that transgender people with experience of sex work or those with intersecting marginalised identities, such as Black transgender women, were most at risk. Additionally, several experts noted that most studies on transgender and non-binary people in Canada were conducted in large metropolitan areas and advocated for studies focusing on these populations in rural communities, about which little is known. No experts were aware of completed research projects corresponding to our inclusion criteria that we had not already identified through our scoping review search.”**

**We have also made some minor changes to the discussion to avoid repetition and expand on some points.**

While revising your submission, please upload your figure files to the Preflight Analysis and Conversion Engine (PACE) digital diagnostic tool, [https://pacev2.apexcovantage.com/](https://can01.safelinks.protection.outlook.com/?url=https%3A%2F%2Ftrack.editorialmanager.com%2FCL0%2Fhttps%3A%252F%252Fpacev2.apexcovantage.com%252F%2F1%2F010f01942bbd24af-4e397bc0-da1f-44a5-99cd-7dcf81b9e37f-000000%2FdxgW_gRbozUD40TCH6eAXar8OY8RDR5rSuG0RqRztw4%3D192&data=05%7C02%7Cjacob.bigio%40mail.mcgill.ca%7C3b582abbb2934c36c5a608dd2be28471%7Ccd31967152e74a68afa9fcf8f89f09ea%7C0%7C0%7C638714974084497827%7CUnknown%7CTWFpbGZsb3d8eyJFbXB0eU1hcGkiOnRydWUsIlYiOiIwLjAuMDAwMCIsIlAiOiJXaW4zMiIsIkFOIjoiTWFpbCIsIldUIjoyfQ%3D%3D%7C0%7C%7C%7C&sdata=gJPNk3bx4KowdujbH%2BDNfMtSoxgNycPeLHr0fQ8Qpdk%3D&reserved=0). PACE helps ensure that figures meet PLOS requirements. To use PACE, you must first register as a user. Registration is free. Then, login and navigate to the UPLOAD tab, where you will find detailed instructions on how to use the tool. If you encounter any issues or have any questions when using PACE, please email PLOS at [figures@plos.org](mailto:figures@plos.org). Please note that Supporting Information files do not need this step.
